# The extracellular matrix component perlecan/HSPG2 regulates radioresistance in prostate cancer cells

**DOI:** 10.3389/fcell.2024.1452463

**Published:** 2024-08-01

**Authors:** Ivana Samaržija, Vasyl Lukiyanchuk, Marija Lončarić, Anja Rac-Justament, Nikolina Stojanović, Ielizaveta Gorodetska, Uğur Kahya, Jonathan D. Humphries, Mahak Fatima, Martin J. Humphries, Ana Fröbe, Anna Dubrovska, Andreja Ambriović-Ristov

**Affiliations:** ^1^ Laboratory for Cell Biology and Signalling, Division of Molecular Biology, Ruđer Bošković Institute, Zagreb, Croatia; ^2^ Laboratory for Epigenomics, Division of Molecular Medicine, Ruđer Bošković Institute, Zagreb, Croatia; ^3^ Helmholtz-Zentrum Dresden-Rossendorf, Institute of Radiooncology-OncoRay, Dresden, Germany; ^4^ OncoRay-National Center for Radiation Research in Oncology, Faculty of Medicine and University Hospital Carl Gustav Carus, Technische Universität Dresden, Helmholtz-Zentrum Dresden-Rossendorf, Dresden, Germany; ^5^ Department of Life Sciences, Manchester Metropolitan University, Manchester, United Kingdom; ^6^ Wellcome Centre for Cell-Matrix Research, Faculty of Biology, Medicine & Health, University of Manchester, Manchester, United Kingdom; ^7^ Department of Oncology and Nuclear Medicine, Sestre Milosrdnice University Hospital Center, School of Dental Medicine, University of Zagreb, Zagreb, Croatia; ^8^ German Cancer Consortium, Partner Site Dresden and German Cancer Research Center, Heidelberg, Germany; ^9^ National Center for Tumor Diseases, Partner Site Dresden: German Cancer Research Center, Heidelberg, Germany; ^10^ Faculty of Medicine and University Hospital Carl Gustav Carus, Technische Universität Dresden, Helmholtz-Zentrum Dresden-Rossendorf, Dresden, Germany

**Keywords:** cell adhesion-mediated radioresistance, prostate cancer, adhesome, matrisome, proteomics, HSPG2, perlecan, biomarker

## Abstract

Radiotherapy of prostate cancer (PC) can lead to the acquisition of radioresistance through molecular mechanisms that involve, in part, cell adhesion-mediated signaling. To define these mechanisms, we employed a DU145 PC model to conduct a comparative mass spectrometry-based proteomic analysis of the purified integrin nexus, i.e., the cell-matrix junction where integrins bridge assembled extracellular matrix (matrisome components) to adhesion signaling complexes (adhesome components). When parental and radioresistant cells were compared, the expression of integrins was not changed, but cell radioresistance was associated with extensive matrix remodeling and changes in the complement of adhesion signaling proteins. Out of 72 proteins differentially expressed in the parental and radioresistant cells, four proteins were selected for functional validation based on their correlation with biochemical recurrence-free survival. Perlecan/heparan sulfate proteoglycan 2 (HSPG2) and lysyl-like oxidase-like 2 (LOXL2) were upregulated, while sushi repeat-containing protein X-linked (SRPX) and laminin subunit beta 3 (LAMB3) were downregulated in radioresistant DU145 cells. Knockdown of perlecan/HSPG2 sensitized radioresistant DU145 RR cells to irradiation while the sensitivity of DU145 parental cells did not change, indicating a potential role for perlecan/HSPG2 and its associated proteins in suppressing tumor radioresistance. Validation in androgen-sensitive parental and radioresistant LNCaP cells further supported perlecan/HSPG2 as a regulator of cell radiosensitivity. These findings extend our understanding of the interplay between extracellular matrix remodeling and PC radioresistance and signpost perlecan/HSPG2 as a potential therapeutic target and biomarker for PC.

## Introduction

Radiotherapy has been used in cancer treatment for more than a century, and it remains one of the primary options for patients. It is estimated that more than 50% of cancer patients will receive radiotherapy during the course of their disease, either as a curative or palliative treatment, and either alone or in combination with other treatment modalities (Weichselbaum et al., 2017). However, resistance to radiotherapy is a frequent occurrence and a major reason for treatment failure.

Localized prostate cancer (PC) is an example of a malignancy that can be cured with radiotherapy or surgery. Currently, both options show equal success (Mottet et al., 2021), and about 38% of patients with localized PC are treated with radiotherapy. Besides the curative treatment in localized disease, radiotherapy is used as an adjuvant, salvage, or palliative therapy in different PC stages. However, depending on the clinical stage, it is estimated that 20%–40% of PC patients will develop recurrence after treatment (Sandhu et al., 2021). Given that PC is among the most common cancers by incidence and mortality, there is an unmet need to elucidate the mechanisms of PC radioresistance and identify biomarkers and potential treatment targets in radioresistant tumors.

Integrins are cell-surface adhesion molecules that bind to extracellular matrix (ECM) ligands (Bachmann et al., 2019). Animal cells are able to sense, adhere to, and remodel their local ECM, thus controlling cell shape, mechanical responsiveness, motility, and fate (Kanchanawong and Calderwood, 2023). Upon ECM ligand engagement, integrins drive the formation of multimolecular scaffolding and signaling structures called integrin adhesion complexes (IACs), which bridge intracellular cytoskeletal and ECM networks at the integrin nexus (Horton et al., 2016). IACs are composed of less than ten to more than hundreds of proteins and differ in a tissue-specific manner in appearance, size, composition, and dynamics. Currently, several major forms of IACs are known, including focal adhesions (FAs), fibrillar adhesions, hemidesmosomes (HDs), and flat clathrin lattices (Zuidema et al., 2020).

Besides malignant cells, the main components of solid cancers include noncancerous cells (vascular cells, cancer-associated fibroblasts, and infiltrating immune cells) located in the tumor microenvironment (TME). The ECM is also part of the TME that controls cancer development and progression (Nikolopoulou et al., 2021). The ECM in the TME is a product of all cells within a tumor and represents a three-dimensional network composed of extracellular organic macromolecules and inorganic components. The ECM mainly consists of collagens (up to 90% of the ECM protein), other glycoproteins (fibronectins, laminins, and elastins), and proteoglycans (Huang et al., 2021). Previously, the ECM was considered a simple structural scaffold; however, it is becoming increasingly evident that it plays essential instructive roles in every aspect of cellular behavior for most developmental and pathogenic processes (Huang et al., 2021).

It is well-established that cell adhesion to the ECM via integrins mediates tumor drug and radiotherapy resistance. Cell adhesion-mediated radioresistance (CAM-RR) mechanisms are complex, diverse, and tumor type-specific (Dickreuter and Cordes, 2017). Changes in the levels of two groups of molecules can lead to the emergence of CAM-RR: (i) adhesion molecules [collectively called the adhesome (Horton et al., 2016)] and (ii) ECM molecules [collectively called the matrisome (Naba et al., 2016)]. The perturbation of either of these two groups of molecules leads to disease. In the last 15 years, due to the development of proteomics, the composition of the matrisome and adhesome has been substantially investigated in cell culture models, enabling the detection of key molecules responsible for the modulation of sensitivity to antitumor drugs (Paradžik et al., 2020; Tadijan et al., 2021) and radiotherapy.

Here, the CAM-RR was studied by using age-matched, paired androgen-independent DU145 parental (DU145 P) and radioresistant (DU145 RR) sublines, which were generated by multiple fractionated irradiation of DU145 cells (Cojoc et al., 2015; Peitzsch et al., 2016). The integrin nexus (IAC and ECM) was isolated from long-term cell cultures of DU145 P and RR sublines and the composition of adhesion signaling and ECM components analyzed using mass spectrometry (MS). Although the expression of integrins was not affected, many adhesome proteins were changed in the DU145 RR cells, and extensive remodeling of ECM was detected in the RR subline. Of the matrisome proteins that were differentially expressed in DU145 P and RR sublines, we focused on those that correlate with biochemical recurrence-free survival (BRFS) in the Cancer Genome Atlas Prostate Adenocarcinoma (TCGA-PRAD) gene expression dataset: perlecan/HSPG2 (heparan sulfate proteoglycan 2), SRPX (sushi repeat-containing protein X-linked), LAMB3 (laminin subunit beta 3), and LOXL2 (lysyl oxidase-like 2). All of these proteins were found to be involved in the regulation of tumor radioresistance. Since these proteins affect the properties of ECM (e.g. its stabilization and cross-linking), we conclude that ECM composition and remodeling is functionally involved in the acquisition of radioresistance in DU145 PC cells. Moreover, these data suggest that perlecan/HSPG2 is a potential therapeutic target among the studied proteins because its knockdown increased the radiosensitivity of both androgen-independent DU145- and androgen-dependent LNCaP-derived radioresistant cells.

## Materials and methods

### Cell cultures

The DU145 and LNCaP PC cell lines were purchased from the American Type Culture Collection (ATCC, United States) and cultured according to the manufacturer’s recommendations in a humidified 37°C incubator supplemented with 5% CO_2_. DU145 cells were maintained in Dulbecco’s modified Eagle’s medium (DMEM; Invitrogen, United States) and LNCaP cells in RPMI-1640 (Sigma-Aldrich, United States), both supplemented with 10% (v/v) fetal bovine serum (FBS; Invitrogen, United States) and 1% L-glutamine (Sigma-Aldrich, United States). The radioresistant sublines (DU145 RR and LNCaP RR) were obtained by serial exposures to irradiation in a previously described manner (Cojoc et al., 2015; Peitzsch et al., 2016). The cell lines have been authenticated using STR profiling within the last 3 years. The cells have been regularly tested for *mycoplasma* contamination, and all experiments were performed with mycoplasma-free cells.

### Isolation of IACs, sample preparation for MS, and data analysis

The integrin nexus (IAC and ECM) were isolated from cells cultivated for 48 h in a previously described manner (Paradžik et al., 2020; Tadijan et al., 2021). Briefly, the crosslinking was performed by incubating the cells with Wang and Richard’s reagent (DTBP, 6 mM, Thermo Fisher Scientific) for 15 min (DU145 cells) and 10 min (LNCaP cells). Isolated integrin nexus proteins were acetone-precipitated and processed for either MS (DU145 P and RR cells) or western blot (WB; DU145 and LNCaP P and RR cells) analysis. Samples were analyzed by LC-MS/MS using an UltiMate 3000 Rapid Separation LC (RSLC, United States) coupled to a Thermo QExactive HF mass detector (Thermo Fisher Scientific, United States) with electrospray ionization. Peptide mixtures were eluted for 60 min. To identify proteins after MS analysis, data were searched against the human Swissprot and Trembl database (12 December 2021) using Mascot (Matrix science, version 2.5.1). Fragment ion tolerance was set to 0.6 Da, while parent ion tolerance was 10 PPM. Scaffold (Proteome Software) was used to refine the identification of proteins further. Total spectral counts were used as a measure of protein abundance. QSpec statistical method (Choi et al., 2008) was used for MS data to measure the significance of differentially identified proteins in DU145 P and DU145 RR cells.

### PPI network formation, functional enrichment analysis, and MS data visualization

Protein-protein interaction networks and functional enrichment analysis of proteins identified with a minimum of four spectral counts in at least two of three biological replicates were constructed in a previously described manner (Paradžik et al., 2020; Tadijan et al., 2021). Annotated matrisome categories were analyzed and visualized using the Matrisome AnalyzeR R package (Petrov et al., 2023).

### SDS-PAGE and western blot (WB) analysis

The preparation of integrin nexus or adhesion protein samples, the SDS-PAGE, and the WB analysis were conducted as described previously (Lončarić et al., 2023). The primary and secondary antibodies are listed in Supplementary Table S1.

### Radiobiological colony formation assay

Radiobiological colony-forming assay was performed as described previously (Cojoc et al., 2015; Peitzsch et al., 2016). 24h after the siRNA transfection, cells were plated at a density of 1,000 cells/well (DU145 P and DU145 RR) or 2,000 cells/well (LNCaP P and LNCaP RR) in 6-well plates in triplicates. For analysis of relative cell radioresistance without siRNA transfection, DU145 and LNCaP, P and RR sublines, were plated in triplicates at 1,000 cells/well in 6-well plates. The following day, cells were irradiated with different doses of X-rays (2, 4, and 6 Gy) using Yxlon Y.TU 320 (200 kV X-rays, dose rate 1.3 Gy/min at 20 mA) filtered with 0.5 mm Cu. A Duplex dosimeter (PTW) was used to measure the absorbed dose. Cells were incubated in a humidified 37°C incubator supplemented with 5% CO_2_ for 10 days. Sham-irradiated cells were used as control. The colonies were fixed with 10% formaldehyde in PBS, stained with a water solution of 0.05% crystal violet, and counted using a stereomicroscope. The plating efficacy (PE) and survival fraction (SF) were calculated as described previously (Cojoc et al., 2015; Peitzsch et al., 2016). The plating efficacy (PE) at 0 Gy (sham) for the same cells cultured simultaneously under identical conditions was used for the normalization (Supplementary Figure S1).

### Analysis of the patient gene expression datasets

The publicly available TCGA PRAD (N = 494) (Sanchez-Vega et al., 2018) and MSKCC PRAD (N = 179) (Taylor et al., 2010) datasets were accessed via cBioportal https://www.cbioportal.org/. The biochemical recurrence-free survival (BRFS) time was used as a clinical endpoint for the Kaplan-Meier survival analysis. The BRFS was determined based on the “Days to PSA” and “Days to biochemical recurrence first” data. The patient groups were defined by the optimal cutoff scan analysis. The raw *p*-value and the best cutoff for the Kaplan-Meier survival analysis were determined using the R2 platform https://hgserver1.amc.nl/cgi-bin/r2/main.cgi.

### Cell adhesion assay

Cells were detached by 1 mM EDTA and seeded at a concentration of 2 × 10^4^ for DU145 cells and 4 × 10^4^ per well for LNCaP cells in a 96-well plate. The cells were allowed to attach for 2 hours and then fixed and stained with crystal violet, and subsequently, the absorbance was measured. The experiment was independently performed three (DU145) or two times (LNCaP), and each replica consisted of at least three technical replicates. For the collagen type I adhesion assay, the cells were handled as described above and plated at the concentrations of 0.5 × 10^4^, 1 × 10^4^ and 2 × 10^4^ for DU145 or 1 × 10^4^, 2 × 10^4^ and 4 × 10^4^ per well for LNCaP cells in a collagen type I -coated 96-well plate (Thermo Fisher Scientific, United States). The relative numbers of viable cells were analyzed using a CellTiter-Glo luminescent assay (Promega, United States) according to the manufacturer’s instructions. The experiment was performed as three independent experiments for DU145 and LNCaP cells, and each biological repeat consisted of at least two technical replicates.

### siRNA transfection

The cells were grown until 60%–80% confluency in a complete medium and transfected with siRNA with Xfect RNA transfection reagent (Takara Bio) according to the manufacturer’s instructions. Cells transfected with unspecific siRNA (Scr siRNA) were used as controls. Cells were harvested 48 h after transfection. The RNA duplexes were synthesized by Eurogentec and used as a pool of two duplexes for each target gene and scrambled (Scr) siRNA. The siRNA sequences are described in Supplementary Table S1.

### RT-qPCR

RNA was isolated by RNeasy Mini kit Plus (Qiagen). Reverse transcription was conducted using the PrimeScript^™^ RT reagent Kit (Takara Bio) according to the manufacturer’s protocol. Quantitative polymerase chain reaction (qPCR) was done using the TB GreenTM Premix Ex TaqTM II (Takara Bio) according to the manufacturer’s recommendation. The qPCR cycling program was as follows: 94°C for 3 min, 40 cycles: 94°C for 15 s, 58°C for 60 s, 72°C for 60 s, followed by a melting curve to 95°C in steps of 0.3°C. qPCR was conducted using the StepOnePlus system (Applied Biosystems). Each analysis was performed with at least three technical replicates. The housekeeper gene RPLP0 or HPRT1 expression was used for data normalization. The primers used in the study are listed in Supplementary Table S1.

### Statistical analysis

The relative cell adhesion, relative gene expression measured by qPCR, and plating efficacy assessed by clonogenic assay were analyzed using paired t-tests. A significant difference between the conditions was defined as *p < 0.05; **p < 0.01; ***p < 0.001. The differences between cell survival curves were analyzed using the Statistical Package for the Social Sciences (SPSS) v23 software by fitting the data into the linear-quadratic model S(D)/S(0) = exp (αD+βD2) using stratified linear regression. The correlation of gene expression levels was calculated using the Pearson correlation coefficient.

## Results

### Analysis of the adhesome composition of DU145 P and RR sublines

**FIGURE 1:**
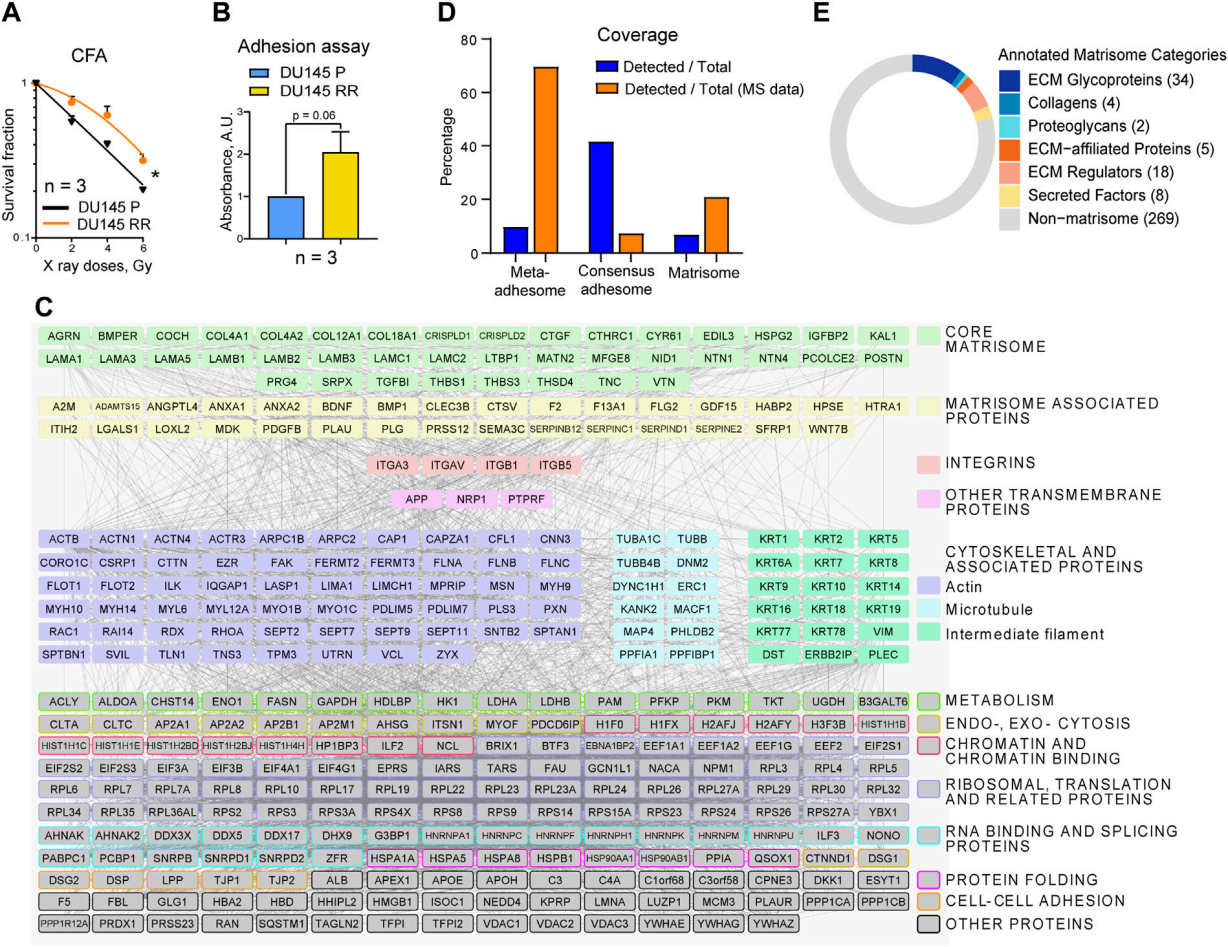
The composition of DU145 adhesion (IACs and ECM) proteins. **(A)** Analysis of the relative cell radiosensitivity of DU145 radioresistant (RR) and parental (P) cells using radiobiological colony formation assay. Data are mean ± SD; *p < 0.05. **(B)** The cell adhesion assay (duration 2 h) reveals that DU145 RR cells show enhanced adhesion properties than DU145 P cells to their own, cell-derived matrix. Data are mean ± SD. **(C)** Protein - protein interaction network of integrin nexus identified through mass spectrometry of proteins isolated from DU145 P cells. Proteins that passed the threshold of spectral count SC ≥ 4 in two out of three replicas in DU145 P cells are shown (340 of 910 proteins in total). The matrisome proteins are categorized according to the MatrisomeDB. In addition, TFPI2, PLAUR, QSOX1 (both P and RR cells), and CCDC80 (only RR cells) were recognized as potential ECM proteins, but they are not included under the matrisome category because this figure aligns with the MatrisomeDB classification. Other proteins were classified according to UniProt and GeneCards databases. Network details: Number of nodes: 340; Number of edges: 4,943; PPI enrichment *p*-value < 1.0e-16. **(D)** The coverage of the meta- and consensus adhesome and matrisome. Orange bars: the percentage of the DU145 IAC proteins belonging to meta and consensus adhesome and matrisome; blue bars: the percentage of the meta and consensus adhesome and matrisome covered in DU145 IAC isolates. **(E)** Matrisome proteins are classified according to the matrisome category to which they belong. The Figure is made by using the Matrisome AnalyzeR R package.

CAM-RR has been described for different tumor types, but the mechanisms operating in PC are not understood. In a search for further potential therapy targets and radiosensitizers in PC, the DU145 P and RR sublines were studied (Figure 1A; Supplementary Figure S1A). Enhanced adhesion properties to the cell-derived matrix of DU145 RR cells were found compared to DU145 P cells (Figure 1B). Therefore, we hypothesized that adhesion proteins (components of the matrisome and adhesome) could affect radioresistance in this PC model.

Initially, the protein isolation protocol to analyze the adhesome and matrisome composition of DU145 P and DU145 RR cells was optimized. Since the cytotoxicity assays for determination of sensitivity to irradiation were performed in cell culture without prior coating with ECM proteins, both adhesion signaling and ECM complexes were isolated in the same manner. Cells were cultured for 48 h, and WB analysis of the marker adhesion components revealed that 15 min of crosslinking with DTBP was optimal. The isolation procedure was performed in triplicate for each cell line, DU145 P and DU145 RR. Samples were analyzed using MS-based proteomics, and spectral counts used as a measure of protein abundance (Supplementary Table S2). Label-free quantification demonstrated good reproducibility. In DU145 P samples, 340 proteins with at least four spectral counts in two out of three replicas were detected, and their composition in the form of a protein-protein interaction network is shown in Figure 1C. The term “meta-adhesome” describes an experimentally defined database consisting of 2,412 proteins observed in at least one of seven fibronectin-initiated IACs proteomes, thus giving an idea of the complexity of the adhesome. A distinct term “consensus adhesome” comprises the 60 proteins identified in at least five datasets (i.e., the most commonly identified, excluding ECM components) and represent both nascent and mature IACs (Horton et al., 2016). The “matrisome” proteome has been defined as a database of ECM and ECM-associated proteins and contains 1,027 proteins (Naba et al., 2016). Figure 1D and Supplementary Table S3 analyze the number of proteins by individual categories. 340 proteins were detected, 237 belonging to the meta-adhesome, 25 to the consensus adhesome, and 71 to the matrisome. Figure 1D depicts the percentage of adhesome and matrisome proteins detected and shows that the consensus adhesome is best represented. Analysis of the percentage of identified proteins in the total dataset shows strong enrichment of meta-adhesome and matrisome (Figures 1D, E). Indeed, gene ontology (GO) analysis (Supplementary Table S4) revealed that the GO terms “extracellular exosome,” “focal adhesion” and “extracellular region” are among the top GO terms in functional enrichment analysis on 340 MS-detected proteins, confirming that the isolations were optimized and contained proteins enriched for adhesome and matrisome components.

As for adhesome proteins, integrin subunits αV, β5, β1, α3, and β3 (sorted by decreasing abundance) were identified, indicating that most probably these cells preferentially use integrins αVβ5, α3β1, αVβ3, and integrin αVβ1 for adhesion. The integrin subunit β3 was just below the threshold of at least four spectral counts in two out of three replicas; thus, it does not appear in Figure 1C. In DU145 P cells, the integrin subunit α6 was not detected; however, in DU145 RR cells, the integrin α6 subunit was detected in only one sample (2 spectra), indicating that at least these cells express integrin α6β1 (which would form FAs) and/or α6β4 (which would form HDs). Since αVβ5 subunits show a relatively high number of spectra in both DU145 P and RR cells, αVβ5 appears to be the preferential integrin heterodimer that these cells use for adhesion and whose expression does not change in DU145 RR cells. Within DU145 P adhesome, all components of the Cortical Microtubule Stabilizing Complex (CMSC) were detected: KANK2, liprin α1 (PPFIA1), liprin β1 (PPFIBP1), microtubule actin crosslinking factor 1 (MACF1), LL5β or pleckstrin homology like domain family B member 2 (PHLDB2), and ELKS/Rab6-interacting/CAST family member 1 (ERC1). Taken together, these results suggest that DU145 P cells form functional FAs, preferentially using αVβ5, αVβ3, and α3β1 and very likely αVβ1, accompanied by CMSC, presumably enabling actin - microtubule crosstalk (Bouchet et al., 2016; Sun et al., 2016; Lončarić et al., 2023).

Previous findings have shown loss of α6β4-dependent hemidesmosomal adhesions in PC cell lines and, especially in DU145 cells, absence of colocalization of integrin α6 and CD151 (Wenta et al., 2022). These results agree with the absence of both HD integrin subunits in DU145 P and the detection of only two spectra in one of the three DU145 RR cell samples (Supplementary Table S2). HDs are among the major forms of IACs that confer stable adhesion of basal epithelial cells to the basement membrane (BM) via integrin α6β4 and are associated with keratin intermediate filaments KRT5 and KRT14. Type I HDs consist of the integrin α6β4, PLEC (isoform 1a), tetraspanin protein CD151, dystonin (DST, BP230 or BPAG1-e), and BP180 (BPAG2 or collagen XVII). In addition, utrophin (UTRN) (Myllymäki et al., 2019) and erbin (ERBIN) (Favre et al., 2001) are β4-interacting proteins found to localize in HDs. Although integrin subunits α6 or β4 were not detected in DU145 P cells by MS, the low expression of hemidesmosomes type I is supported by the detection of several other HD proteins like plectin (PLEC), dystonin (DST), utrophin (UTRN), erbin (ERBIN), keratins 5 (KRT5) and 14 (KRT14) but not CD151. Therefore, we conclude that DU145 P cells preferentially use FAs but may also form low levels of HDs for adhesion in long-term cell culture.

### Analysis of the integrin αVβ5 IACs composition

In our recent publication (Paradžik et al., 2020), we determined the adhesome of integrin αVβ5, which forms FAs in the melanoma cell line MDA-MB-435S. We established that talins 1 and 2 (TLN1 and 2), α-actinins 1 and 4 (ACTN1 and 4), filamins A and B (FLNA and B), and vinculin (VCL) were the key components of integrin αVβ5 FAs. Indeed, in the DU145 P adhesome, we found all these major FA proteins except TLN2 (Figure 1C; Supplementary Table S2). Similarly, Jin et al. (Jin et al., 2014) demonstrated very low expression of TLN2 in metastatic PC cell line PC3. Indeed, TLN2 knockout mice are viable and fertile (Debrand et al., 2012). In line with this, we have recently shown that TLN2 knockdown does not destroy FAs and cannot compensate for the TLN1 knockdown-induced loss of FAs (Lončarić et al., 2023). Among the cytoskeleton proteins, the most represented group was actin-binding proteins, while the microtubule and intermediate filament cytoskeleton proteins were less abundant but not absent. We have previously shown that KN motif and ankyrin repeat domain-containing protein 2 (KANK2) is a key molecule linking integrin αVβ5 FAs to MTs (Paradžik et al., 2020) and that KANK2 within Cortical Microtubule Stabilising Complex (CMSC) functionally interacts with TLN2 in regulation of actin-MT crosstalk. However, which talin isoforms will bind to which KANK isoform is likely to be cell-specific (Lončarić et al., 2023).

### Analysis of the matrisome composition of DU145 P and RR sublines

The role of the matrisome was predicted bioinformatically by using the characteristic domain-based organization of ECM proteins (Naba et al., 2016). It is evident from Figures 1C, D, E and Supplementary Tables S3, S5, and S6 that DU145 P cells secrete a complex ECM, which can be sub-classified into core matrisome and matrisome-associated proteins categories (Supplementary Table S5). Seventy-one proteins, or 20.9% of all proteins above the threshold detected in MS analysis, belong to the matrisome category. Furthermore, 6.9% of all matrisome proteins (Naba et al., 2016) were detected in the DU145 matrisome. Figure 1C shows that DU145 P cells secrete a variety of basement membrane (BM) proteins, including laminins (LAMA1, LAMA3, LAMA5, LAMB1, LAMB2, LAMB3, LAMC1, and LAMC2, suggesting the presence of laminins-111, -121, -332, -311, -321, -511, 521, and 522), collagen IV (COL4A1 and COL4A2), collagen XVIII (COL18A1) and collagen XII (COL12A1). Major BM proteins agrin (AGRN) and perlecan/heparan sulfate proteoglycan 2 (HSPG2) and both nidogens (NID1 in DU145 and DU145 RR cells, and NID2 in DU145 RR cells) were also present. Furthermore, netrins 1 and 4 (NTN1 and NTN4), which are frequently incorporated in BMs, were also detected. Other proteins that were found in DU145 matrisome include ECM glycoproteins such as collagen cleavage peptidase procollagen C-endopeptidase enhancer 2 (PCOLCE2), latent transforming growth factor beta binding protein 1 (LTBP1), integrin binding periostin (POSTN), thrombospondins 1 and 3 (THBS1 and THBS3) and vitronectin (VTN). Of ECM-affiliated proteins, semaphorin 3C (SEMA3C) and annexins A1 and A2 (ANXA1, ANXA2) were detected. Among the ECM regulators, ADAMTS zinc metalloendopeptidase 15 (ADAMTS15), bone morphogenetic protein 1 (BMP1), and collagen cross-linking protein lysyl oxidase-like 2 (LOXL2) were found. Secreted factors such as angiopoietin-like 4 (ANGPTL4), growth differentiation factor 15 (GDF15), and midkine (MDK) were also detected (Supplementary Table S5). Taken together, these data indicate that ECM composition in DU145 cells consists of core components, secreted remodeling enzymes, and soluble factors that potentially regulate cell fate.

### Comparison of DU145 P and DU145 RR IAC and ECM composition

**FIGURE 2:**
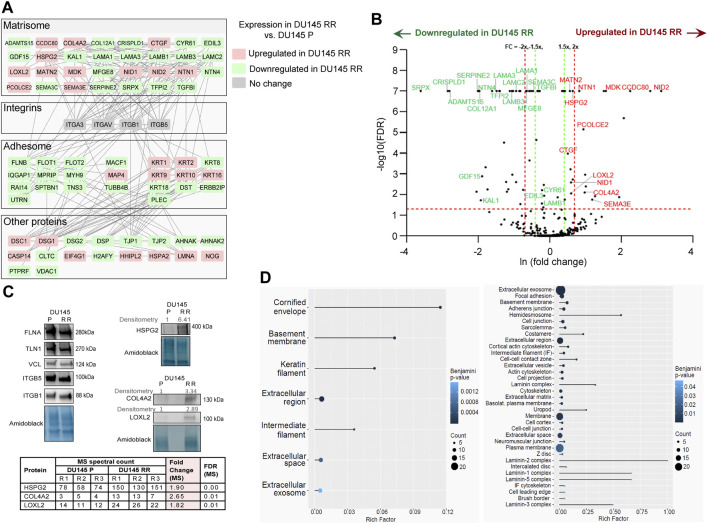
Differentially expressed adhesion (IACs and ECM) proteins (DEPs) in DU145 RR compared to DU145 P cells. **(A)** Protein-protein interaction network of DEPs and **(B)** volcano plot of proteins that passed the threshold SC ≥ 4 in two out of three replicas in DU145 P and/or RR cells (419 proteins). Matrisome proteins, including CCDC80 and TFPI2, are highlighted. Dashed lines represent 0.05 FDR (statistical significance after QSpec analysis)-value (horizontal line) and 1.5 (green) and 2-fold (red) change cut-off values (vertical lines). **(C)** WB validation of selected adhesion proteins from DU145 P and DU145 RR cells. Forty-eight hours after seeding, integrin nexus proteins were isolated, and WB analysis was performed. Fold change for selected DEPs obtained by WB (densitometry) is calculated by using ImageJ and amido-black as a reference. Selected proteins whose expression does not change are also included in WB validation. The table shows the spectral counts for selected DEPs obtained by MS. Fold change (MS) and FDR (MS) in DU145 RR versus DU145 P cells are calculated by the QSpec statistical method obtained on MS data. “R1,” “R2,” and “R3” stand for three biological repeats. **(D)** Enrichment analysis obtained on differentially expressed proteins. The left panel shows an analysis of proteins upregulated in DU145 RR cells (n = 26), and the right panel shows an analysis of downregulated proteins (n = 46). The analysis was performed using the online tool DAVID (Database for Annotation, Visualization, and Integrated Discovery), and the results were visualized using the ggplot2 R package. All terms for which the Benjamini adjusted *p*-value is <0.05 are shown. The Rich Factor is the ratio between the number of detected proteins in a term and the total number of proteins in that term. The circle size corresponds to the number of detected proteins in a term (the Count).

In DU145 RR cells, 26 upregulated and 46 downregulated proteins were found compared to parental DU145 P cells (Figures 2A, B). Analysis of the gene expression data for DU145 P and RR cells (GEO accession number: GSE134499) demonstrated that most of these proteins have the same expression trends at the levels of mRNA (Supplementary Figures S1B, S1C), suggesting that their deregulation in RR cells occurs mainly at the transcriptional level. The MS data were validated using WB. FA proteins identified in MS by a large number of spectra, i.e., FLNA, TLN1, vinculin, and integrin subunits β5 and β1, were selected. As observed in MS analysis, the expression of these proteins was not altered in radioresistant cells. By contrast, three ECM proteins with increased abundance in MS: perlecan/HSPG2, a classical BM protein COL4A2 (Kuo et al., 2012), and copper-dependent amine oxidase LOXL2 that promotes collagen crosslinking (Csiszar, 2001), were upregulated as assessed by WB (Figure 2C). Supplementary Tables S6, S7, and S8 provide a complete list of proteins with changed expression in DU145 RR, along with their enrichment analysis and functional annotation. The results of the enrichment analysis are visualized in Figure 2D. Database for Annotation, Visualization, and Integrated Discovery (DAVID) enrichment analysis of the upregulated proteins (Figure 2D; Supplementary Tables S7, S8) resulted in several top terms, including, “cornified envelope,” “basement membrane,” and “extracellular region.” Analysis of the downregulated proteins resulted in the top terms “extracellular exosome,” “focal adhesion,” and “hemidesmosome.” The 72 proteins with changed expression include 29 in the matrisome category (Naba et al., 2016). A comparison of DU145 P and DU145 RR adhesome and matrisome composition suggests that extensive ECM composition and remodeling occurred during the radioresistance development.

Among the upregulated proteins in radioresistant DU145 RR cells a significant enrichment of the GO term “cornified envelope” includes different members of the keratin gene family (type II cytokeratins KRT1 and KRT2; type I (acidic) cytokeratins KRT10 and KRT16), and two proteins of the desmosome cell-cell junction desmoglein 1 (DSG1) and desmocollin1 (DSC1). Even more interesting is the group of proteins found under the GO term “basement membrane” including its main structural elements like COL4A2, CCDC80 (coiled-coil domain containing 80), glycoproteins NID1 and NID2, proteoglycan perlecan/HGSP2, ECM regulator LOXL2 and a poorly studied protein NTN1 known to be expressed in prostate tumors (Latil et al., 2003). As many as 14 proteins are gathered under the GO term “extracellular region” and in addition to previously mentioned COL4A2, HSPG2, NID1 and NID2, NTN1, CCDC80, and KRT1, includes bone morphogenetic protein 4 inhibitor noggin (NOG), procollagen C-endopeptidase enhancer-2 (PCOLCE2) that enhances the catalytic activity of BMP1, SEMA3E whose overexpression was shown to affect PC cell adhesion and migration (Blanc et al., 2011), growth factor MDK shown to be involved in PC drug resistance (Saikia et al., 2023), hedgehog interacting protein-like 2 (HHIPL2), cellular communication network factor 2 (CCN2) and matrilin 2 (MATN2), indicating the extensive extracellular matrix remodeling. GO terms “keratin filament” and “intermediate filament” are also high on the list of upregulated GO terms; however, their genes (mainly keratins) are already covered by the “cornified envelope” category.

Among the downregulated proteins in DU145 RR cells, the significant enrichment of the GO term “extracellular exosome” contains as many as 27 proteins. Here are the typical exosome proteins like cystein-rich secretory protein LCCL domain containing 1 (CRISPLD1), the core matrisome proteins COL12A1, LAMB1, LAMA3, MFGE8, SEMA3C, EGF-like repeats, and discoidin domains 3 (EDIL3) and GDF15. We also found proteins associated with endocytosis, either clathrin-mediated, i.e., clathrin heavy chain (CLTC), or caveolae-mediated, i.e., flotillin 1 and 2 (FLOT1 and FLOT2). We detected several proteins known to be found in FAs, like actin-associated proteins (FLNB), IQ motif containing GTPase activating protein 1 (IQGAP1), myosin heavy chain 9 (MYH9), and spectrin beta non-erythrocytic (SPTBN1). Other identified proteins include those associated with intermediate filaments, keratins KRT8 and KRT18, PLEC and UTRN, then protein tyrosine phosphatase receptor type F (PTPRF), and the giant AHNAK nucleoprotein (AHNAK), which is readily found in IAC isolates (Paradžik et al., 2020; Tadijan et al., 2021). Still it is not known whether it represents its structural component. We also found two desmosomal proteins, desmoplakin (DSP), DSG2, and finally, a macroH2A.1 histone (MACROH2A1) and mitochondrial constituent voltage-dependent anion-selective channel protein 1 (VDAC1). Interestingly, among the top enrichment of GO terms are “focal adhesions,” “basement membrane,” “adherens junction” and “hemidesmosomes.” A careful examination of the proteins in the group “focal adhesions” showed overlap with proteins covered by the term “extracellular exosome” and does not include many other proteins typically found in FAs (integrins αV, β5, β1, α3, and β3, TLN1, ACTN4). Therefore, we conclude that there was no change in the amount of FAs in radioresistant DU145 RR cells, and that the proteins that show a differential expression are most likely those that the cell secretes by exocytosis. Considering that HDs are composed of a smaller number of proteins, many of which are found in exosomes (PLEC, DST, and LAMA3 but also HD-associated ERBIN), it is difficult to conclude about the amount of HDs. The fact that KRT5 and KRT14 were detected by a high number of spectra (Supplementary Table S2) and that we did not find changes in their abundance indicates that there was no significant change in the amount of possibly present HDs. Conversely, in DU145 RR cells, we saw downregulation of all laminin-332 subunits (LAMA3, LAMB3, and LAMC2), the main HD ligand, which could indicate a change in HD function. A decrease in the function of HDs in DU145 RR cells would be in line with the recently published study, which suggests that the disassembly of α6β4-mediated HDs promotes tumorigenesis in PTEN-negative PC (Wenta et al., 2022). In conclusion, since integrin α6 (ITGA6) and β4 (ITGB4) were not detected by MS while laminin subunit LAMB3, which is specific for laminin 332, is detected by a high number of spectra and less abundant in DU145 RR cells, we hypothesized that cell ECM, rather than the IACs, might be the source of molecules potentially conferring radioresistant phenotype.

### ECM proteins perlecan (HSPG2), SRPX, LAMB3, and LOXL2 contribute to radioresistance

**FIGURE 3:**
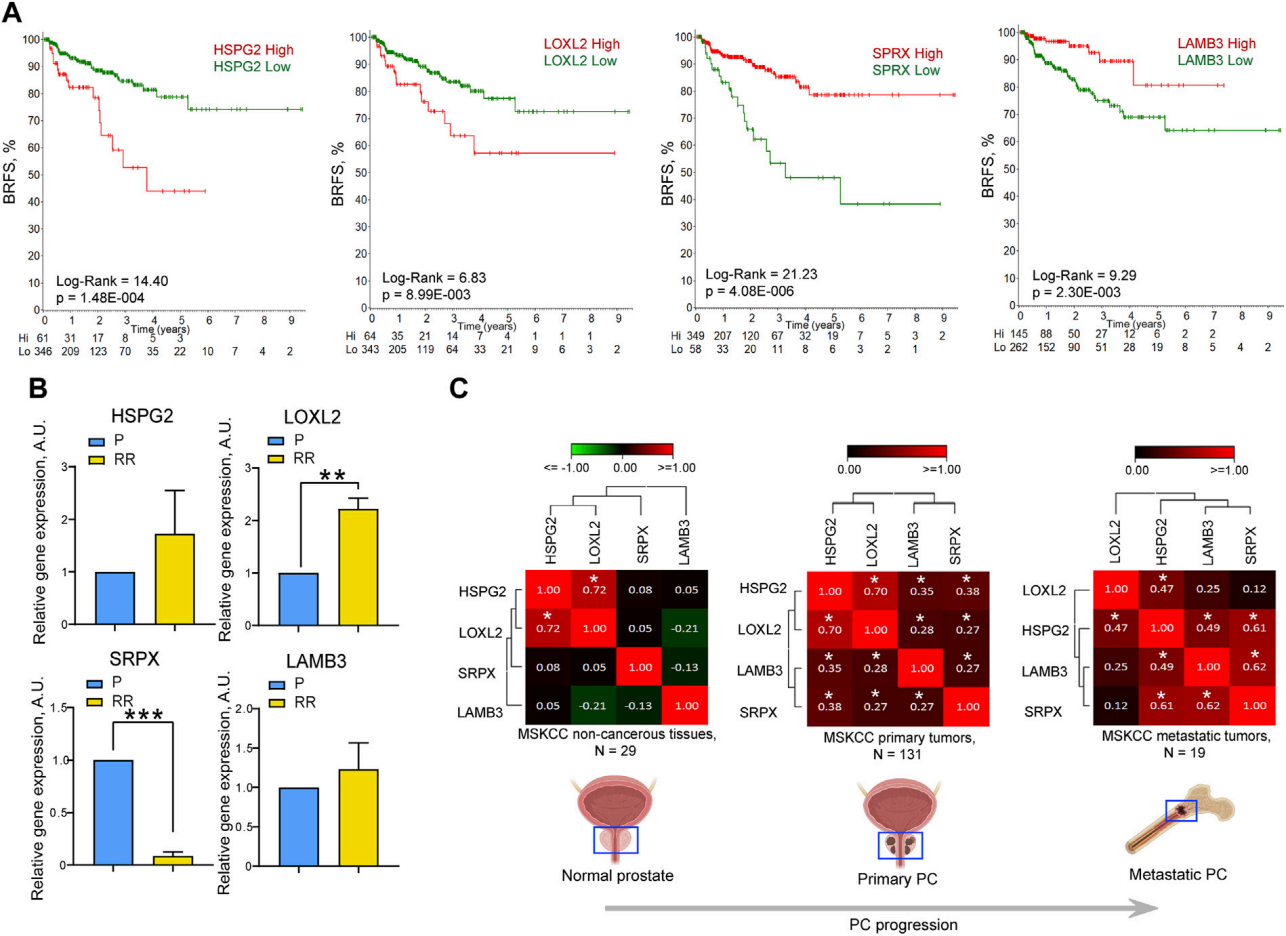
The expression levels of HSPG2, LOXL2, LAMB3, and SRPX genes correlate with the clinical outcomes and cell radioresistance. **(A)** The Kaplan-Meier analyses of the association of HSPG2, LOXL2, LAMB3, and SRPX gene expression and biochemical recurrence-free survival (BRFS) in the TCGA PRAD PC gene expression dataset (N = 407). The stratification of patients into “high” and “low” groups according to gene expression was obtained using the online tool R2 Platform (https://hgserver1.amc.nl/cgi-bin/r2/main.cgi?option=kaplan_main). The numbers on the *x*-axis represent time in years. Numbers at risk are added to the bottom of each graph. **(B)** RT-qPCR analysis of the relative HSPG2, LOXL2, SRPX, and LAMB3 mRNA expression in DU145 P and RR cells. N = 3; Error bars = SD; **p < 0.01; ***p < 0.001. **(C)** Correlation of HSPG2, LOXL2, SRPX, and LAMB3 mRNA expression levels in normal tissues (MSKCC dataset, n = 29), primary tumors (MSKCC dataset, n = 131), and metastatic tumors (MSKCC dataset, n = 19). The correlation of gene expression levels was calculated using the Pearson correlation coefficient; *p < 0.05.

The ECM is a complex and dynamic interconnected network of macromolecules that surround cells and provide a scaffold to maintain tissue structure. In cancer, the ECM within the tumor microenvironment plays an integral role in cancer initiation, progression, and response to treatments (Krisnawan et al., 2020). It is evident from Figures 1, 2 that DU145 P and RR cells secrete plentitude of ECM proteins with potentially essential roles in radioresistance, many of which are differentially expressed. To select proteins that could be potential biomarkers of PC clinical outcomes and radioresistance, the gene expression and clinical parameters from The Cancer Genome Atlas (TCGA) prostate adenocarcinoma (PRAD) dataset (N = 493) were analyzed to determine the correlation of differentially expressed genes and survival of patients with PC (Supplementary Figure S2). Perlecan/HSPG2 and LOXL2 gene expression significantly correlated with worse biochemical recurrence-free survival (BRFS) (Figure 3A), whereas SRPX and LAMB3 had a significant correlation with favorable BRFS. Perlecan/HSPG2 is the major structural constituent of BMs of most endothelial and epithelial cells that participates in the various stages of cancer progression by regulating interactions between cells and signaling molecules (Elgundi et al., 2020) and is upregulated in prostate TME (Warren et al., 2014). LOXL2 is overexpressed in PC, and knockdown of the LOXL2 gene markedly inhibited the migration and invasion of PC cells (Kato et al., 2017). In addition, cancer-associated fibroblast-derived LOXL2 is an essential mediator of intercellular communication within the prostate TME and is a potential therapeutic target (Nguyen et al., 2019). SRPX is downregulated in PC compared to normal prostate or benign prostate hyperplasia (BPH) and is one of seven candidate genes identified that exhibit reduced expression and increased promoter methylation, a pattern characteristic of tumor suppressors (Kamdar et al., 2019). Finally, laminin-332 is a component of the ECM that contributes to the BM architecture and is downregulated in PC (Hao et al., 2001). ZEB1, a regulator of EMT, has been shown to repress the expression of laminin-332 and its receptor, β4 integrin, in PC cells (Drake et al., 2010).

Analysis of the BRFS of patients who received radiotherapy showed a significant correlation with the SRPX and LOXL2 expression levels, whereas, for the LAMB3 and perlecan/HSPG2, the results were not statistically significant, which can be attributed to the low number of patients (N = 38) (Supplementary Figure S3). The mRNA expression level of these genes was analyzed in the DU145 model and both genes associated with worse BRFS, perlecan/HSPG2, and LOXL2 were found to be highly expressed in the DU145 RR cell line. In contrast, the SRPX gene associated with a more favorable outcome was downregulated in RR cells compared to their parental counterpart (Figure 3B). Contrary to the proteomics data, the downregulation of LAMB3 mRNA expression was not observed in DU145 RR cells, which may be explained by a potential effect of posttranslational modifications on protein stability.

Furthermore, the interplay between these four genes increases during tumor development, as evidenced by increased correlation between the expression levels of perlecan/HSPG2 and LAMB3 or perlecan/HSPG2 and SRPX in primary PC and metastatic PC compared to normal tissues in the MSKCC dataset (N = 179) (Taylor et al., 2010) (Figure 3C). Knockdown of LAMB3 in both DU145 P and RR cell lines was associated with significant downregulation of the perlecan/HSPG2 gene expression, suggesting that the interplay between these genes could also be seen in our *in vitro* models (Supplementary Figure S4). These findings suggest that the expression levels of these four ECM genes could be used as a potential predictor of PC patient outcomes and tumor radioresistance.

### Validation of the functional role of perlecan/HSPG2, SRPX, LAMB3, and LOXL2 in PC radioresistance

**FIGURE 4:**
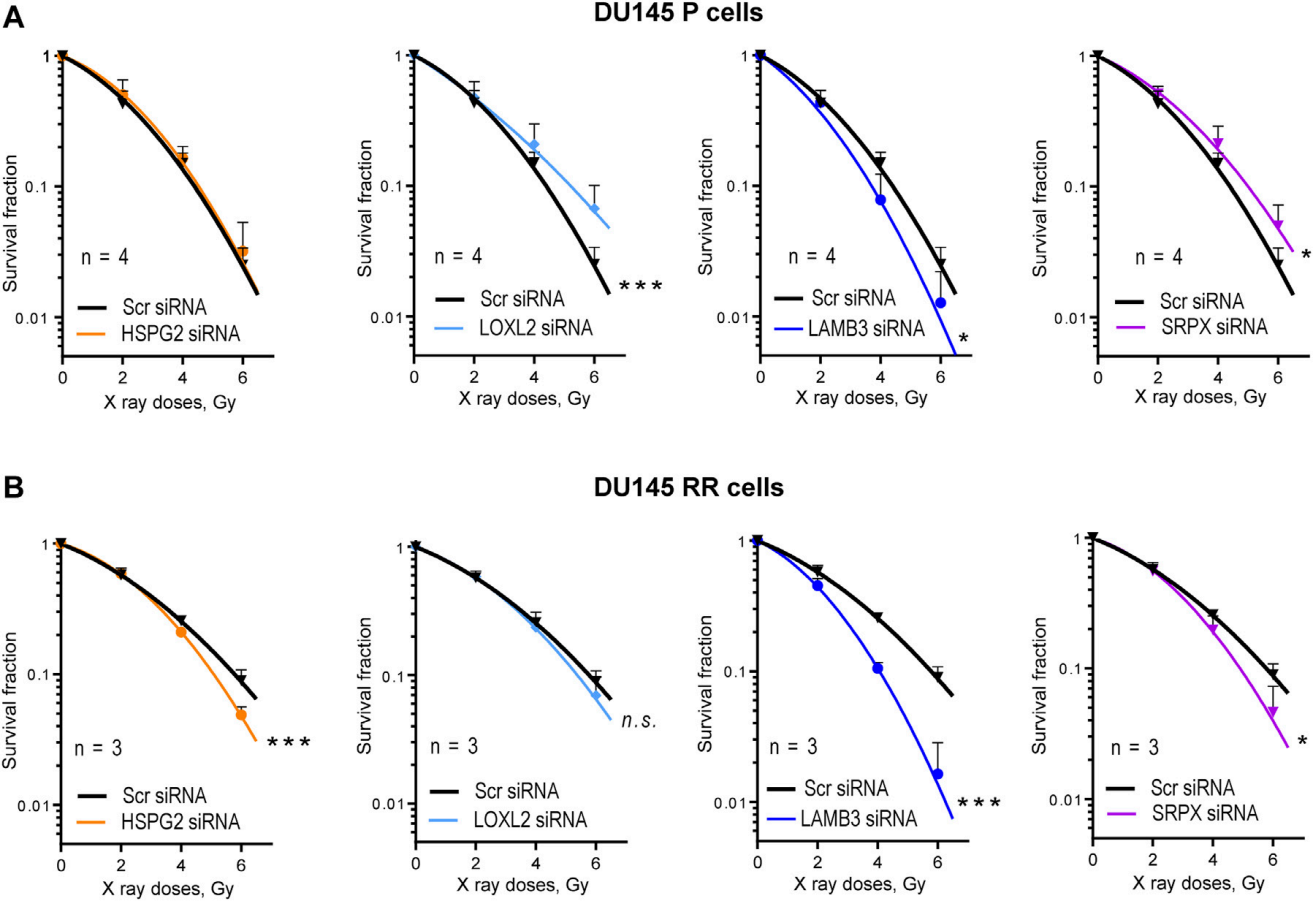
Radiobiological colony formation assay (CFA). **(A)** CFA for the DU145 parental (P) cells after siRNA-mediated knockdown of HSPG2, LOXL2, LAMB3, or SRPX. Cells transfected with scrambled (Scr) siRNA were used as controls. Data are mean ± SD; *p < 0.05; ***p < 0.001. **(B)** CFA for the DU145 radioresistant (RR) cells after siRNA-mediated knockdown of HSPG2, LOXL2, LAMB3, or SRPX. Cells transfected with scrambled siRNA (Scr siRNA) were used as controls. Data are mean ± SD; *p < 0.05; ***p < 0.001.

The potential role of four ECM proteins in regulating cell radioresistance was further analyzed by radiobiological clonogenic analyses after the siRNA-mediated knockdown of each target gene. This analysis demonstrated that 3 out of 4 genes play different roles in regulating the radiosensitivity in P and RR cells. Knockdown of perlecan/HSPG2, LOXL2, and SRPX led to the radiosensitization of RR cells, whereas only knockdown of LAMB3 expression resulted in PC cell radiosensitization in both P and RR cell lines (Figure 4; Supplementary Figure S5). Among the analyzed genes, only knockdown of the perlecan/HSPG2 led to the specific radiosensitization of RR cells. However, in contrast to the knockdown of LOXL2 and SRPX, this was not associated with increased radioresistance in P cells.

**FIGURE 5:**
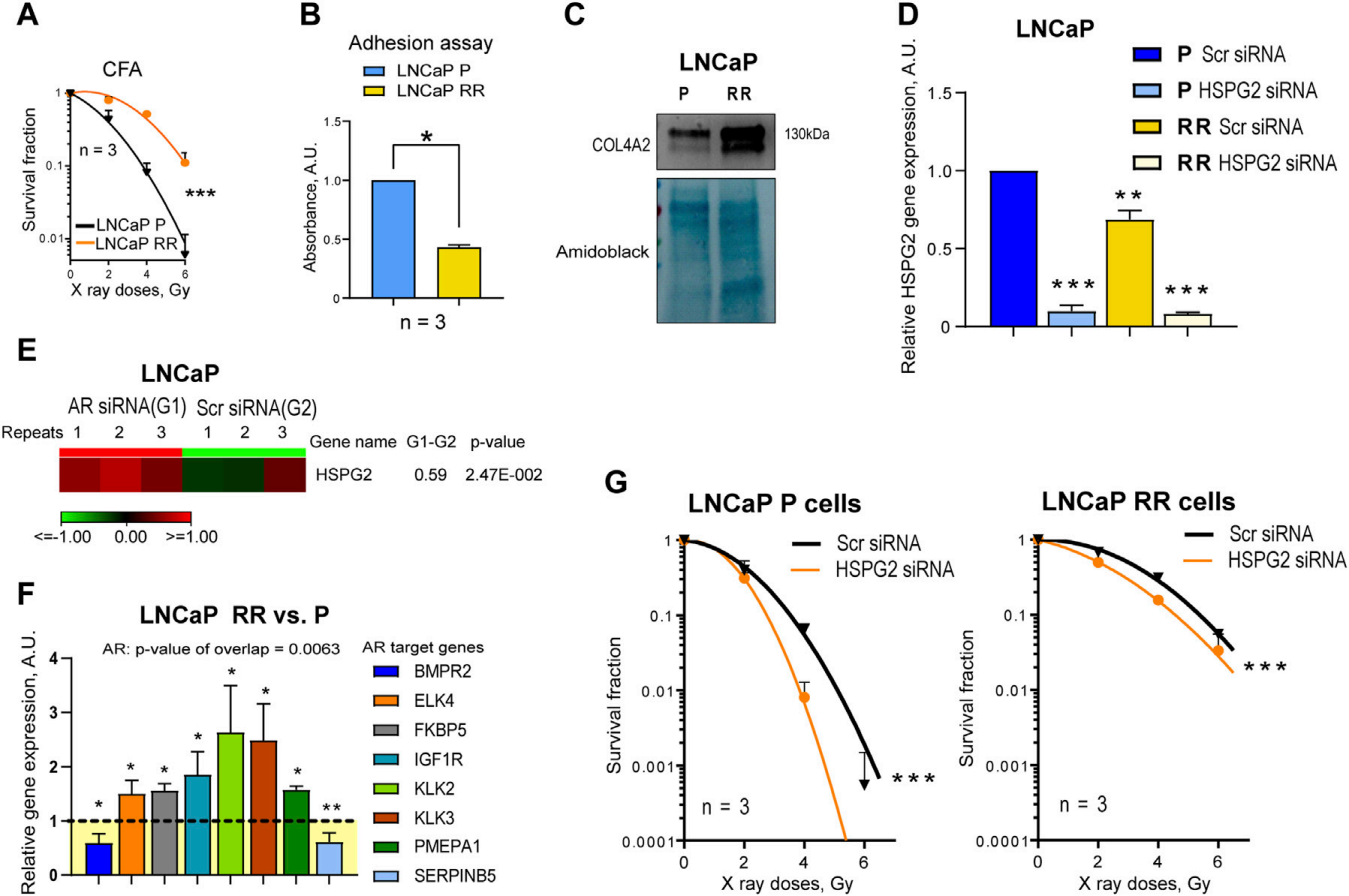
Validation of the finding using androgen-responsive LNCaP parental (P) and radioresistant (RR) cells. **(A)** Analysis of the relative cell radiosensitivity of LNCaP radioresistant (RR) and parental (P) cells using radiobiological colony formation assay. Data are mean ± SD; ***p < 0.001. **(B)** The cell adhesion assay reveals that LNCaP P cells show enhanced adhesion properties to their own, cell derived matrix than LNCaP RR cells. Data are mean ± SD; *p < 0.05. **(C)** Western blot analysis of ECM protein COL4A2 in LNCaP P and LNCaP RR cells. Forty-eight hours after seeding, integrin nexus proteins were isolated, and WB analysis was performed. **(D)** RT-qPCR analysis of the relative perlecan/HSPG2 expression in LNCaP P and RR cells. Cells were transfected with perlecan/HSPG2 siRNA or scrambled (Scr) siRNA as a control. N = 3; Error bars = SD; **p < 0.01; ***p < 0.001. **(E)** RNA seq analysis of the relative perlecan/HSPG2 expression in LNCaP P cells. Cells were transfected with AR siRNA or Scr siRNA as a control as described earlier (Gorodetska et al., 2024). N = 3. **(F)** The Ingenuity pathway analysis (IPA) has identified AR as one of the upstream transcriptional regulators activated in the LNCaP RR cells. The comparative gene expression of the AR-responsive genes in LNCaP P and LNCaP RR cells (GSE134499) N = 3; Error bars = SD; *p < 0.05; **p < 0.01. **(G)** CFA on the LNCaP P and RR cells after siRNA-mediated knockdown of perlecan/HSPG2. Cells transfected with Scr siRNA were used as controls. Data are mean ± SD; ***p < 0.001.

To validate these findings in androgen-independent DU145 cells using additional PC models, radiobiological clonogenic survival was analyzed in the androgen-responsive LNCaP P and RR cells (Figure 5A; Supplementary Figure S6A) established in our previous studies (Cojoc et al., 2015; Peitzsch et al., 2016). Cell adhesion to the cell-derived matrix analysis (Figure 5B) showed the opposite result to that seen in DU145 cells, i.e., radioresistant LNCaP RR cells adhered less than parental LNCaP P cells. However, COL4A2 protein was detected with increased abundance in isolated integrin nexus from LNCaP RR cells in a similar manner to that observed for DU145 cells (Figure 5C), suggesting some similarities between the cell models. Of note, in contrast to DU145 RR cells, LNCaP RR cells possessed significantly higher adhesion to the collagen type I than their P counterparts (Supplementary Figure S6B). In part, that can be explained by an elevated expression of the IGF-1R, which is known to induce integrin-mediated PC adhesion to collagen (Siech et al., 2022) (Supplementary Figure S6C). Perlecan/HSPG2 and LOXL2 were not detected by WB analysis of isolated integrin nexus, indicating that expression of these molecules in LNCaP is lower than in DU145. This differs from the results of Datta et al. (Datta et al., 2006), who showed by RT-qPCR and WB that the highest perlecan/HSPG2 expression is present in LNCaP cells compared to several androgen-independent cells, including DU145. However, our WB method only detects the perlecan/HSPG2 protein secreted by cells and incorporated into the cell-derived matrix. Our results of RT-qPCR analysis showed that radioresistant LNCaP RR cells express less perlecan/HSPG2-specific mRNA than parental LNCaP P cells (Figure 5D), which is different from that observed in the DU145 cell model. This discrepancy can be explained by the negative regulation of perlecan/HSPG2 by androgen receptor (AR) in LNCaP cells (Figure 5E). The Ingenuity pathway analysis has identified AR as one of the upstream transcriptional regulators activated in the LNCaP RR cells compared to the P cells (Figure 5F). Nevertheless, functional radiobiological clonogenic assay revealed that similar to DU145 RR cells, both LNCaP P and RR cells were radiosensitized by perlecan/HSPG2 knockdown (Figure 5G; Supplementary Figure S6D), indicating that perlecan/HSPG2 could be a potential therapeutic target in androgen-sensitive and androgen-independent PC cells.

## Discussion

Although targeting different pathways has been explored as a radiosensitizer in PC in preclinical studies, androgen deprivation therapy is currently the only treatment modality that synergizes with radiation in clinical trials and is an accepted approach for patients with high-risk disease (Denham et al., 2011; Zapatero et al., 2015; Mottet et al., 2021). Clinical studies confirm that combining radiotherapy with targeted therapies holds promise for designing more effective PC treatment strategies. Previous publications on PC cells showed that acquiring radioresistance is a complex process involving multiple molecular mechanisms. For example, it was demonstrated that prostate progenitor cells with an enhanced DNA repair capacity and activation of epithelial-mesenchymal transition (EMT) contribute to radioresistance in these cells (Cojoc et al., 2015). Moreover, epigenetic reprogramming (Peitzsch et al., 2016), glutamine catabolism, autophagy (Mukha et al., 2021), retinoid-dependent gene transcription (Gorodetska et al., 2024), and cell plasticity (Schwarz et al., 2022) were shown to play a role. Among the radioresistance mechanisms, CAM-RR is another mediator that cells can rely on upon exposure to irradiation. Since our initial data indicated the increased (>2-fold) capability of adhesion of DU145 RR cells in comparison to DU145 P cells, we focused on the influence of proteins involved in adhesion (IAC and ECM) on radioresistance.

Comparative MS-based proteomic analysis of the integrin nexus isolated from DU145 P and RR cells revealed 72 differentially regulated proteins. Among these we identified four genes, perlecan/HSPG2, LAMB3, LOXL2, and SRPX, that were significantly associated with clinical outcomes in patients with PC: perlecan/HSPG2 and LOXL2 gene expression correlated with worse BRFS, whereas SRPX and LAMB3 correlated with better BRFS. We also confirmed that perlecan/HSPG2 and LOXL2 are highly expressed in the DU145 RR cells compared to their radiosensitive counterpart, DU145 P cells.

All these four proteins are reported to be involved in PC pathogenesis and regulation of tumor radioresistance. The perlecan/HSPG2 gene encodes the perlecan/HSPG2 protein, which is among the major components of the BM. Increased perlecan/HSPG2 expression has been observed in many different tumors, including PC [reviewed in (Theocharis and Karamanos, 2019)]. However, the perlecan/HSPG2 in a dense ECM in different tumors *in vivo* is accumulated not only by tumor cells but also by various stromal cells and immune cells (Elgundi et al., 2020). Perlecan/HSPG2 also accumulates in the desmoplastic stroma of PC in response to cytokines (Warren et al., 2014). In PC sections, it co-localizes with MMP-7 at tissue boundaries, and the release of active perlecan/HSPG2 fragments can regulate essential PC cell functions such as adhesion and invasion (Grindel et al., 2014; Grindel et al., 2016). *In vitro*, prostate fibroblasts modify the cellular organization by secreting perlecan/HSPG2 in spheroid cocultures with PC3 and DU145 PC cells (Ojalill et al., 2020). The importance of perlecan/HSPG2 in PC is well established. Datta et al. (Datta et al., 2006) demonstrated that perlecan/HSPG2, a candidate gene for the CAPB locus with familial risk of brain and PC, is a component of Sonic Hedgehog (SHH) signaling, and its expression in PC tissues correlates with a high Gleason score and rapid cell proliferation. Perlecan/HSPG2 gene over-expression promotes tumor cell growth, chemoresistance, migration, and invasion *in vivo* and *in vitro* (Theocharis and Karamanos, 2019). The targeted reduction of perlecan/HSPG2 in the bone-targeted PC line C4-2B xenografts (Wu et al., 1994) growing in mice reduced tumor growth and vascularization (Savorè et al., 2005). This data is consistent with our finding on the potential role of perlecan/HSPG2 as a biomarker of PC progression and its high expression in more malignant, radioresistant DU145 RR cells.

The second selected candidate, LAMB3, also belongs to a family of BM proteins. It was shown that the knockdown of LAMB3 in nasopharyngeal carcinoma decreased radioresistance (Zhuang et al., 2023), which is not in line with our finding of its reduced expression in DU145 RR cells, but it completely fits to our functional studies. The third selected protein, LOXL2, belongs to the lysyl oxidase gene family, and we found its upregulation in DU145 RR cells. This protein catalyzes the first step in the formation of crosslinks in collagens and elastin and is, therefore, involved in ECM assembly and the regulation of the PC tumor microenvironment (Nguyen et al., 2019). Its role in PC radioresistance has been previously suggested (Xie et al., 2019). Finally, SRPX (sushi repeat-containing protein X-linked) is predicted to be an ECM structural constituent involved in cell adhesion. In PC, lower expression of SRPX was shown to correlate with poorer recurrence-free survival in patients and significantly lower expression of SRPX in high-risk Gleason scores of eight tumors compared to low- or intermediate-risk tumors was noted (Kamdar et al., 2019). This observation is in line with our study, which found decreased expression of SRPX in the ECM of more malignant, radioresistant DU145 RR cells.

Our previous studies suggest that the course of irradiation given to PC cell models induces major genetic and epigenetic changes (Peitzsch et al., 2016). In addition, in such an experimental system that in some way mimics what happens in tumors during exposure to radiotherapy, it is not expected that radioresistance will arise due to increased or decreased expression of one or even a group of genes, but is rather a result of the deregulation of many genes cumulatively contributing to the PC pathogenesis and radioresistance. Of note, we confirmed a correlation between the expression levels of perlecan/HSPG2 and LAMB3 or HSPG2 and SRPX and found that it increases during tumor progression.

Therefore, we analyzed the functional role of each of these ECM proteins in regulating cell radioresistance. Genes overexpressed in RR cells are more attractive therapeutic targets because inhibiting their expression or biological functions could sensitize cells to radiotherapy. However, at the same time, it is important that the knockdown of the same gene does not have the opposite effect in the parental cells. The only target protein that meets these criteria is perlecan/HSPG2, which is upregulated in DU145 RR. Perlecan/HSPG2 knockdown in DU145 RR cells made them more sensitive to irradiation, whereas its knockdown in DU145 P did not affect their radiosensitivity. We also validated the radiosensitizing effect of perlecan/HSPG2 knockdown in the androgen-sensitive LNCaP P and RR cell models. This observation indicates that perlecan/HSPG2 may be a potential target independent of the androgen responsiveness of the used cell line models. Together with a correlation of perlecan/HSPG2 with clinical outcomes, this would suggest that perlecan/HSPG2 is a potential target and biomarker in PC. An additional discussion of the role of LAMB3, LOXL2, and SRPX in tumor radioresistance is included in the Supplementary Material.

Of note, PC cells show increased tropism to the bone (La Manna et al., 2019). Perlecan/HSPG2 is an essential ECM component involved in the growth responses of metastatic PC cells to heparin-binding growth factors deposited in local and metastatic microenvironments (Savorè et al., 2005). Therefore, targeting perlecan/HSPG2 may not only increase the sensitivity of PC to radiotherapy but may also block bone metastases and associated morbidity. An ideal biomarker for PC should be non-invasively assessed, inexpensive, highly sensitive, and specific. For anatomical reasons, urine is enriched in prostatic secretions. Perlecan/HSPG2 is secreted and can be easily detected in urine samples (Lima et al., 2022). Investigating perlecan/HSPG2 as a potential therapeutic target and biomarker for PC not only holds promise for enhancing radiotherapy effectiveness but also offers a pathway towards mitigating the burden of bone metastases and improving patient outcomes. Further clinical validation of perlecan/HSPG2 as a tissue-based and non-invasive biomarker would be important to validate its role as a marker of PC progression and radioresistance.

## Data Availability

The datasets presented in this study can be found in online repositories. The names of the repository/repositories and accession number(s) can be found below: https://www.ebi.ac.uk/pride/archive/, PXD052368.
