# Supplementary material for: The extracellular matrix component perlecan/HSPG2 regulates radioresistance in prostate cancer cells
Source: Front Cell Dev Biol. 2024 Aug 1;12:1452463. doi: 10.3389/fcell.2024.1452463 (PMC11325029; doi:10.3389/fcell.2024.1452463)
Supplement: Supplementary file 1 [file Presentation1.pdf]

Supplementary Material

Supplementary Tables

Table S1. List of antibodies, siRNA oligonucleotides, and primers used in this study.

Table S5. DU145 matrisome proteins are classified according to MatrisomeDB. The numbers in parentheses denote the number of proteins found in DU145 integrin adhesions in that category out of the total number of proteins that belong to a certain category.

Table S6. Differentially expressed proteins in DU145 RR compared to DU145 P cells. The roles of the proteins were deduced from the GeneCards database (www.genecards.org). FDR = statistical significance after QSpec analysis, FC = fold change in DU145 RR vs. DU145 P cells.

Table S1. List of antibodies, siRNA oligonucleotides and primers used in this study.

**Antibodies used in the western blot experiment**				
**Antibody**	**Catalogue number**	**Manufacturer**	**Dilution**	**Species**
Filamin A (FLNA)	sc-17749	Santa Cruz Biotechnology, USA	1:500	Mouse
Integrin β5 (ITGB5)	CST 3629	Cell Signaling Technology, USA	1:1000	Rabbit
Talin 1 (TLN1)	MCA4770GA	Bio-Rad, USA	1:1000	Mouse
Vinculin (VCL)	ab129002	Abcam, USA	1:6000	Rabbit
Integrin β1 (ITGB1)	MAB1965-I	Merck Millipore, Germany	1:1000	Mouse
Collagen, type IV, alpha 2 (COL4A2)	AP51015PU-N	Origene, USA	1:200	Rabbit
Lysyl Oxidase Like 2 (LOXL2)	ab277807	Abcam, USA	1:500	Rabbit
Perlecan (HSPG2)	sc-377219	Santa Cruz Biotechnology, USA	1:1000	Mouse

**siRNA oligonucleotides used for knockdown of gene expression**		
**Gene**	**siRNA name**	**mRNA target sequence**
HSPG2	siHSPG2-13308<br>siHSPG2-4325	GCCCGACAAUGUCGAGUAU<br>GGUGGGAAGUUGCGAUACA
LOXL2	siLOXL2-2402<br>siLOXL2-1291	CGAGGUUGCAGAAUCCGAU<br>GGACCGUCUGCGACGACAA
SRPX	siSRPX-1429<br>siSRPX-826	AGAUGGUCCUACAAGCCGA<br>CUUGCAAAUUUCGAGUUAA
LAMB3	siLAMB3-2738<br>siLAMB3-320	GGAAGAUGUCAGACGCACA<br>CUACUACAGUCACCGAGUA
Scrambled (Scr)	siScr-MCR<br>siScr-EGFP	GCAGCUAUAUGAAUGUUGU<br>GGCUACGUCCAGGAGCGCA

**Primers used for RT-qPCR**	
**Gene**	**Sequence (5’->3’)**
HSPG2	F 5'- CCAAATGCGCTGGACACATTC-3'<br>R 5'- CGGACACCTCTCGGAACTCT-3'
LOXL2	F 5'- AGGACATTCGGATTCGAGCC-3'<br>R 5'- CTTCCTCCGTGAGGCAAAC-3'
SRPX	F 5'- TGCTCCAGCGACGGTGATA-3'<br>R 5'- TGTGGACACAATGAGGAGTCT-3'
LAMB3	F 5'- GCAGCCTCACAACTACTACAG-3'<br>R 5'- CCAGGTCTTACCGAAGTCTGA-3'
RPLP0	F 5'- CTCAACATCTCCCCCTTCTCCTT-3'<br>R 5'- TGATGCAACAGTTGGGTAGCC-3'
HPRT1	F 5'- CTTTGCTGACCTGCTGGATTAC-3'<br>R 5'- TTGCGACCTTGACCATCTTTG-3'

Table S5. DU145 ECM proteins classified according to MatrisomeDB. The numbers in parentheses denote the number of proteins found in DU145 integrin adhesions in that category out of the total number of proteins that belong to a certain category.

**Category**		**Proteins**
**Core matrisome** (40/274)	**ECM glycoproteins** (34/195)	AGRN, BMPER, COCH, CRISPLD1, CRISPLD2, CTGF, CTHRC1, CYR61, EDIL3, IGFBP2, KAL1, LAMA1, LAMA3, LAMA5, LAMB1, LAMB2, LAMB3, LAMC1, LAMC2, LTBP1, MATN2, MFGE8, NID1, NTN1, NTN4, PCOLCE2, POSTN, SRPX, TGFBI, THBS1, THBS3, THSD4, TNC, VTN
	**Collagens** (4/44)	COL4A1, COL4A2, COL12A1, COL18A1
	**Proteoglycans** (2/35)	HSPG2, PRG4
**Matrisome associated proteins** (31/753)	**ECM-affiliated proteins** (5/171)	ANXA1, ANXA2, CLEC3B, LGALS1, SEMA3C
	**ECM regulators** (18/238)	A2M, ADAMTS15, BMP1, CTSV, F2, F13A1, HABP2, HPSE, HTRA1, ITIH2, LOXL2, PLAU, PLG, PRSS12, SERPINB12, SERPINC1, SERPIND1, SERPINE2
	**Secreted factors** (8/344)	ANGPTL4, BDNF, FLG2, GDF15, MDK, PDGFB, SFRP1, WNT7B

Table S6. Differentially expressed proteins in DU145 RR in comparison to DU145 P cells. The roles of the proteins were deduced from the GeneCards database (www.genecards.org). FDR = statistical significance after QSpec analysis, FC = fold change in DU145 RR vs. DU145 P cells.

Protein	FDR	FC	Role
NID2	0.000001	22.49	Basement membrane (BM) protein. Cell-adhesion protein that binds collagens I and IV and laminin and may be involved in maintaining the structure of the BM.
HSPA2	0.000001	16.56	Molecular chaperone implicated in a wide variety of cellular processes.
CCDC80	0.000001	9.44	Predicted to act upstream of or within extracellular matrix organization.
NOG	0.000002	7.93	Inhibitor of bone morphogenetic proteins (BMP) signaling.
CASP14	0.013381	6.97	Non-apoptotic caspase involved in epidermal differentiation.
MDK	0.000001	4.80	Secreted protein that functions as cytokine and growth factor.
HHIPL2	0.000001	4.51	Predicted to enable catalytic activity. Predicted to be located in extracellular region.
DSC1	0.012142	3.54	Component of intercellular desmosome junctions.
SEMA3E	0.017671	3.27	Plays an important role in signaling. Mediates reorganization of the actin cytoskeleton. Promotes FA disassembly and inhibits adhesion of endothelial cells to the ECM.
EIF4G1	0.004096	2.74	Part of the EIF4F complex which facilitates the recruitment of mRNA to the ribosome.
DSG1	0.001103	2.69	Component of intercellular desmosome junctions.
COL4A2	0.007991	2.65	One of the six subunits of type IV collagen, the major structural component of BM.
PCOLCE2	0.000007	2.56	Binds to the C-terminal propeptide of types I and II procollagens and may enhance the cleavage of that propeptide by BMP1.
NTN1	0.000001	2.28	Thought to be involved in axon guidance and cell migration during development.
HSPG2	0.000001	1.90	Integral component of BMs. A large multidomain proteoglycan that binds to and cross-links many ECM components and cell-surface molecules.
MAP4	0.001863	1.90	Non-neuronal microtubule-associated protein. Promotes microtubule assembly.
LOXL2	0.008814	1.81	Extracellular copper-dependent amine oxidase that catalyzes the first step in the formation of crosslinks in collagens and elastin.
NID1	0.002625	1.81	Sulfated glycoprotein widely distributed in BMs and tightly associated with laminin. Also binds to collagen IV and perlecan. It probably has a role in cell-ECM interactions.
KRT16	0.000602	1.79	Epidermis-specific type I keratin that plays a key role in skin. IF protein.
MATN2	0.000001	1.75	Involved in matrix assembly.
CTGF	0.000105	1.66	Major connective tissue mitoattractant secreted by vascular endothelial cells.
KRT9	0.000001	1.53	Type I keratin 9, an intermediate filament chain.
LMNA	0.017870	1.52	Component of the nuclear lamina.
KRT1	0.000001	1.47	Member of the keratin gene family. Intermediate filament (IF) protein.
KRT2	0.000001	1.41	Member of the keratin gene family. IF protein.
KRT10	0.013738	1.24	Member of the type I (acidic) cytokeratin family. IF protein.
EDIL3	0.011446	0.85	An integrin ligand. Promotes adhesion of endothelial cells.
PLEC	0.003445	0.85	Interlinks IFs with microtubules and microfilaments and anchors IFs to desmosomes or hemidesmosomes (HDs).
SPTBN1	0.042044	0.83	Actin crosslinking and molecular scaffold protein that links the plasma membrane to the actin cytoskeleton.
CYR61	0.006133	0.80	Interacts with several integrins and with heparan sulfate proteoglycan.
LAMB1	0.027465	0.80	Laminins are the major noncollagenous constituent of BMs.
AHNAK	0.000001	0.75	Large structural scaffold protein, may be required for neuronal cell differentiation.
TGFBI	0.000001	0.74	RGD-containing protein that binds to type I, II and IV collagens; role in cell adhesion.
MYH9	0.000001	0.71	Cellular myosin that appears to play a role in cytokinesis, cell shape, secretion & capping.
CLTC	0.000023	0.69	The major protein of the polyhedral coat of coated pits and vesicles that are involved in the intracellular trafficking of receptors and endocytosis of a variety of macromolecules.
SEMA3C	0.000001	0.63	Secreted glycoprotein that belongs to the family of neuronal guidance cues.
FLNB	0.000001	0.59	Connects cell membrane constituents to the actin cytoskeleton. May promote orthogonal branching of actin filaments.
KRT18	0.000223	0.57	Type I IF chain keratin 18; together with its filament partner keratin 8, are perhaps the most commonly found members of the IF gene family.
IQGAP1	0.000001	0.56	Plays a crucial role in regulating the dynamics and assembly of the actin cytoskeleton.
LAMA1	0.000001	0.53	The laminins are a family of ECM glycoproteins that have a heterotrimeric structure.

			These proteins make up a major component of the BM and have been implicated in a wide variety of biological processes including cell adhesion, differentiation, migration, signaling, neurite outgrowth and metastasis.
MFGE8	0.000001	0.52	Plays an important role in the maintenance of intestinal epithelial homeostasis.
TUBB4B	0.005958	0.47	The major constituent of microtubules
DSP	0.029439	0.44	Major high molecular weight protein of desmosomes. Anchors IFs to desmos. plaques.
PTPRF	0.000001	0.44	Member of the protein tyrosine phosphatase (PTP) family; possible cell adhes. receptor.
UTRN	0.000001	0.43	May play a role in anchoring the cytoskeleton to the plasma membrane. Integrin α6β4 recruits UTRN to Madin-Darby canine kidney cells (MDCK)-HDs (hemidesmosomes).
VDAC1	0.002510	0.39	Forms a channel through the mitochondrial outer and the plasma membrane.
LAMC2	0.000001	0.39	Laminins, a family of ECM glycoproteins, are the major noncollagenous constituent of BMs. Implicated in a wide variety of biological processes.
DST	0.000001	0.36	Cytoskeletal linker protein. Acts as an integrator of IFs, actin and microtubule cytoskeleton networks. Required for anchoring either IFs to the actin cytoskeleton in neural and muscle cells or keratin-containing IFs to hemidesmosomes in epithelial cells.
MACF1	0.000001	0.35	Forms bridges between different cytoskeletal elements.
DSG2	0.001107	0.35	Component of intercellular desmosome junctions. Involved in the interaction of plaque proteins and IFs mediating cell-cell adhesion.
LAMB3	0.000001	0.35	Belongs to a family of BM proteins. The LAMB3 gene encodes the laminin β3 chain, which is a unique component of laminin-332.
KRT8	0.000001	0.33	Member of the type II keratin family.
TJP2	0.005556	0.31	Plays a role in tight junctions and adherens junctions.
H2AFY	0.007328	0.30	Variant histone H2A which replaces conventional H2A in a subset of nucleosomes.
ERBIN	0.002495	0.30	Acts as an adapter for the receptor ERBB2, in epithelia. Found in hemidesmosomes.
FLOT2	0.000031	0.28	May act as a scaffolding protein within caveolar membranes.
TFPI2	0.000001	0.25	May play a role in the regulation of plasmin-mediated matrix remodeling.
TNS3	0.000001	0.25	May play a role in actin remodeling. Involved in the dissociation of the integrin-tensin-actin complex.
FLOT1	0.000001	0.24	May act as a scaffolding protein within caveolar membranes.
LAMA3	0.000001	0.23	Binding to cells via a high affinity receptor, laminin is thought to mediate the attachment, migration and organization of cells into tissues during embryonic development by interacting with other ECM components.
NTN4	0.000001	0.20	Functions in various biological processes including axon guidance, tumorigenesis, and angiogenesis. Netrins are laminin-related proteins.
MPRIP	0.005716	0.18	Targets myosin phosphatase to the actin cytoskeleton. Enables cadherin binding activity.
TJP1	0.000484	0.17	TJP1, TJP2, and TJP3 are closely related scaffolding proteins that link tight junction (TJ) transmembrane proteins such as claudins, junctional adhesion molecules, and occludin to the actin cytoskeleton.
GDF15	0.001301	0.15	Secreted ligand of the TGF-beta superfamily of proteins.
ANOS1	0.019118	0.15	Gene Ontology annotations related to this gene include heparin binding and ECM structural constituent.
AHNAK2	0.000001	0.14	A large nucleoprotein.
COL12A1	0.000001	0.14	A member of the FACIT (fibril-associated collagens with interrupted triple helices) collagen family. Found in association with type I collagen, an association that is thought to modify the interactions between collagen I fibrils and the surrounding matrix.
SERPINE2	0.000001	0.13	Serine protease inhibitor with activity toward thrombin, trypsin, and urokinase.
RAI14	0.007321	0.13	Plays a role in actin regulation at the ectoplasmic specialization, a type of cell junction.
ADAMTS15	0.000001	0.06	Metalloprotease which has proteolytic activity against the proteoglycan VCAN.
CRISPLD1	0.000001	0.06	Involved in face morphogenesis. Located in extracellular exosome.
SRPX	0.000001	0.03	Predicted to be an ECM structural constituent. Predicted to be involved in cell adhesion.

Supplementary Figures

Figure S1. A) Plating efficiencies (PE, %) in the radiobiological colony formation assay for DU145 parental (P) and radioresistant (RR) cells. B) Analysis of the gene expression data for DU145 P and RR cells (GEO accession number: GSE134499) for the integrin nexus proteins downregulated in DU145 RR compared to DU145 P, as shown in Figure 2B. C) Analysis of the gene expression data for DU145 P and RR cells (GEO accession number: GSE134499) for the integrin nexus proteins upregulated in DU145 RR compared to DU145 P, as shown in Figure 2B.

Figure S2. The Kaplan-Meier analyses of the association of the gene expression and biochemical recurrence-free survival (BRFS) in the TCGA PRAD PC gene expression dataset (N = 407; left insert) and separately on the patients that received radiotherapy, RT (n=38; right insert). The stratification of patients into 'high' and 'low' groups according to gene expression was obtained using the online tool R2 Platform (https://hgserver1.amc.nl/cgi-bin/r2/main.cgi?option=kaplan_main). The numbers on the x-axis represent time in years. Numbers at risk are added to the bottom of each graph.

Figure S3. The Kaplan-Meier analyses of the association of the perlecan/HSPG2, SRPX, LOXL2, and LAMB3 gene expression and biochemical recurrence-free survival (BRFS) in the TCGA PRAD PC gene expression dataset, a sub-cohort of patients who received radiotherapy, RT (N = 38).

Figure S4. RT-qPCR analysis of the relative perlecan/HSPG2, SRPX, LOXL2, and LAMB3 gene expression in DU145 P and DU145 RR cells. Cells were transfected with perlecan/HSPG2, SRPX, LOXL2, and LAMB3 target siRNA or with scrambled (Scr) siRNA as a control. N = 3; Error bars = SD; $*p < 0.05$; $**p < 0.01$; $***p < 0.001$.

Figure S5. Plating efficiencies (PE, %) in DU145 parental (P) and radioresistant (RR) cells were analyzed after siRNA-mediated knockdown on perlecan/HSPG2, LOXL2, LAMB3, or SRPX. Cells transfected with scrambled (Scr) siRNA were used as controls. Data are mean ± s.d. There are no statistically significant differences in plating efficiency among the different conditions (n.s.).

Figure S6. A) Plating efficiencies (PE, %) in the radiobiological colony formation assay for LNCaP parental (P) and radioresistant (RR) cells. Data are mean ± SD; $*p < 0.05$. B) Cell adhesion to the collagen type I – coated plates. DU145 P, DU145 RR, LNCaP P, and LNCaP RR were plated in the 96 well plates in at least two replicates using the indicated cell number plated per well. Data are mean ± SD; $*p < 0.05$. C) Relative IGF1R gene expression in LNCaP P and LNCaP RR cells. Gene expression profiling was performed as described in *Cojoc et al., 2015* and *Seifert et al*., *2019* (GSE134499). Data are mean ± SD; $*p < 0.05$. D) Plating efficiencies (PE, %) in the radiobiological colony formation assay for LNCaP cells. PE for the LNCaP parental (P) and radioresistant (RR) cells were analyzed after siRNA-mediated knockdown on perlecan/HSPG2. Cells transfected with scrambled (Scr) siRNA were used as controls. Data are mean ± SD; $*p < 0.05$; $**p < 0.01$.

Figure S1

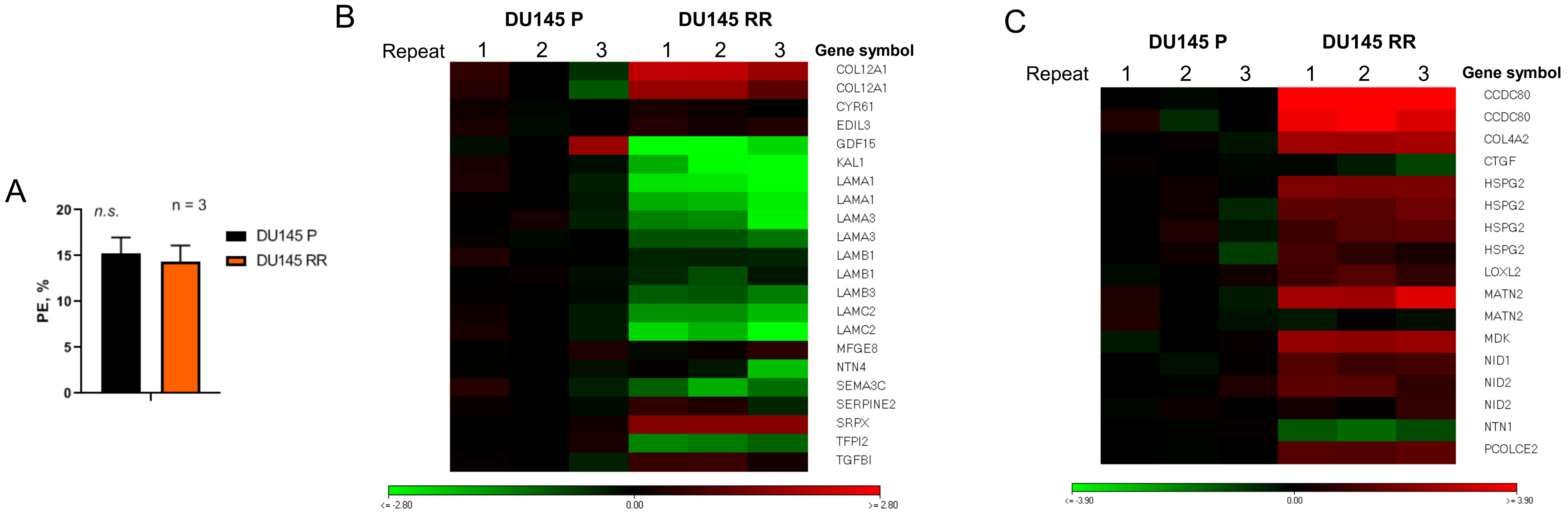
A
n.s.
n = 3
PE, %
20
15
10
5
0
DU145 P
DU145 RR
B
DU145 P
DU145 RR
Repeat 1 2 3 1 2 3
Gene symbol
COL12A1
COL12A1
CYR61
EDIL3
GDF15
KAL1
LAMA1
LAMA1
LAMA3
LAMA3
LAMB1
LAMB1
LAMB3
LAMC2
LAMC2
MFGE8
NTN4
SEMA3C
SERPINE2
SRPX
TFPI2
TGFBI
<= -2.80
0.00
>= 2.80
C
DU145 P
DU145 RR
Repeat 1 2 3 1 2 3
Gene symbol
CCDC80
CCDC80
COL4A2
CTGF
HSPG2
HSPG2
HSPG2
HSPG2
LOXL2
MATN2
MATN2
MDK
NID1
NID2
NID2
NTN1
PCOLCE2
<= -3.90
0.00
>= 3.90

Figure S2

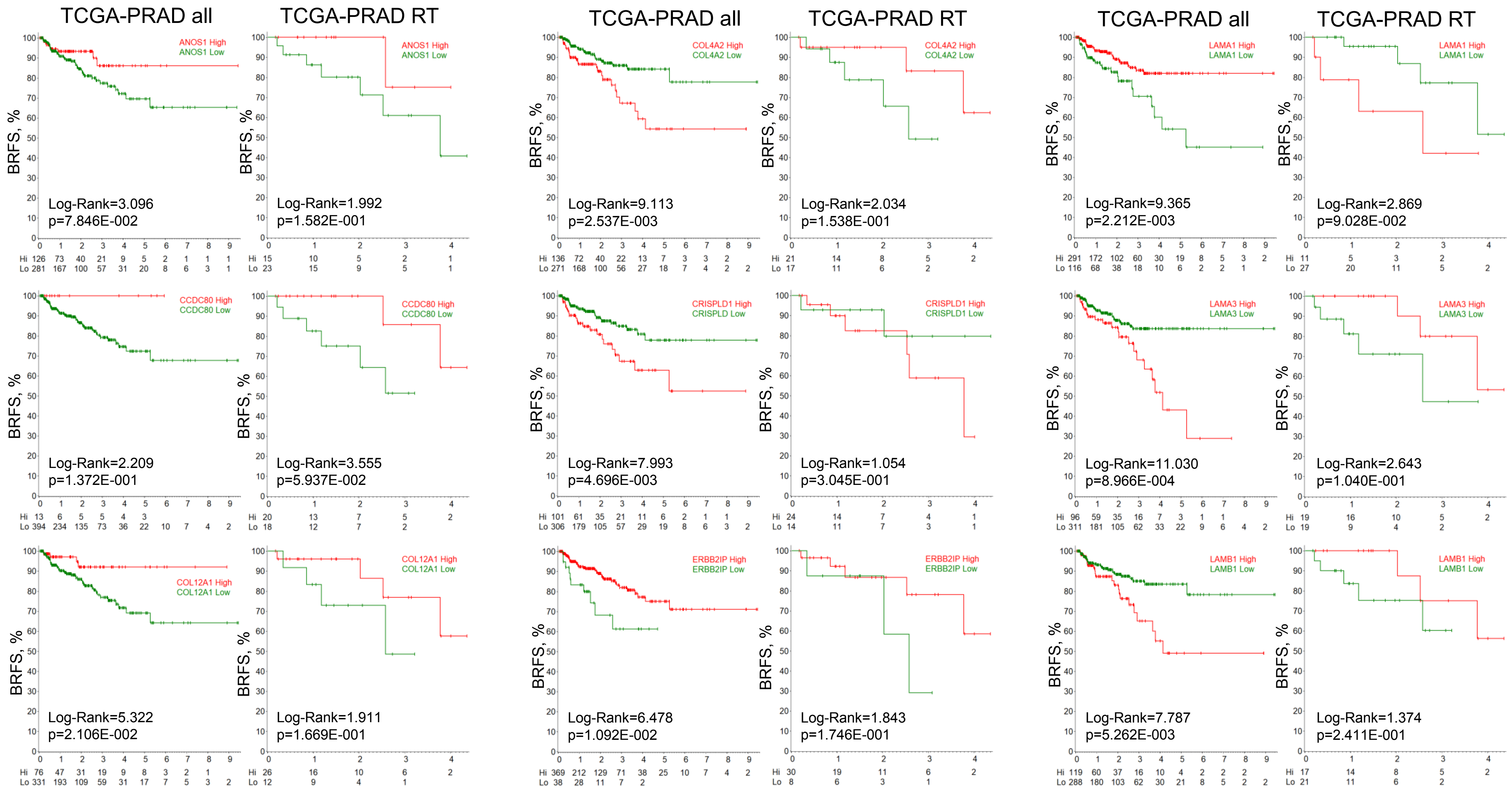
TCGA-PRAD all
TCGA-PRAD RT
TCGA-PRAD all
TCGA-PRAD RT
TCGA-PRAD all
TCGA-PRAD RT
BRFS, %
ANOS1 High
ANOS1 Low
Log-Rank=3.096
p=7.846E-002
ANOS1 High
ANOS1 Low
Log-Rank=1.992
p=1.582E-001
COL4A2 High
COL4A2 Low
Log-Rank=9.113
p=2.537E-003
COL4A2 High
COL4A2 Low
Log-Rank=2.034
p=1.538E-001
LAMA1 High
LAMA1 Low
Log-Rank=9.365
p=2.212E-003
LAMA1 High
LAMA1 Low
Log-Rank=2.869
p=9.028E-002
CCDC80 High
CCDC80 Low
Log-Rank=2.209
p=1.372E-001
CCDC80 High
CCDC80 Low
Log-Rank=3.555
p=5.937E-002
CRISPLD1 High
CRISPLD Low
Log-Rank=7.993
p=4.696E-003
CRISPLD1 High
CRISPLD1 Low
Log-Rank=1.054
p=3.045E-001
LAMA3 High
LAMA3 Low
Log-Rank=11.030
p=8.966E-004
LAMA3 High
LAMA3 Low
Log-Rank=2.643
p=1.040E-001
COL12A1 High
COL12A1 Low
Log-Rank=5.322
p=2.106E-002
COL12A1 High
COL12A1 Low
Log-Rank=1.911
p=1.669E-001
ERBB2IP High
ERBB2IP Low
Log-Rank=6.478
p=1.092E-002
ERBB2IP High
ERBB2IP Low
Log-Rank=1.843
p=1.746E-001
LAMB1 High
LAMB1 Low
Log-Rank=7.787
p=5.262E-003
LAMB1 High
LAMB1 Low
Log-Rank=1.374
p=2.411E-001

Figure S2, continued

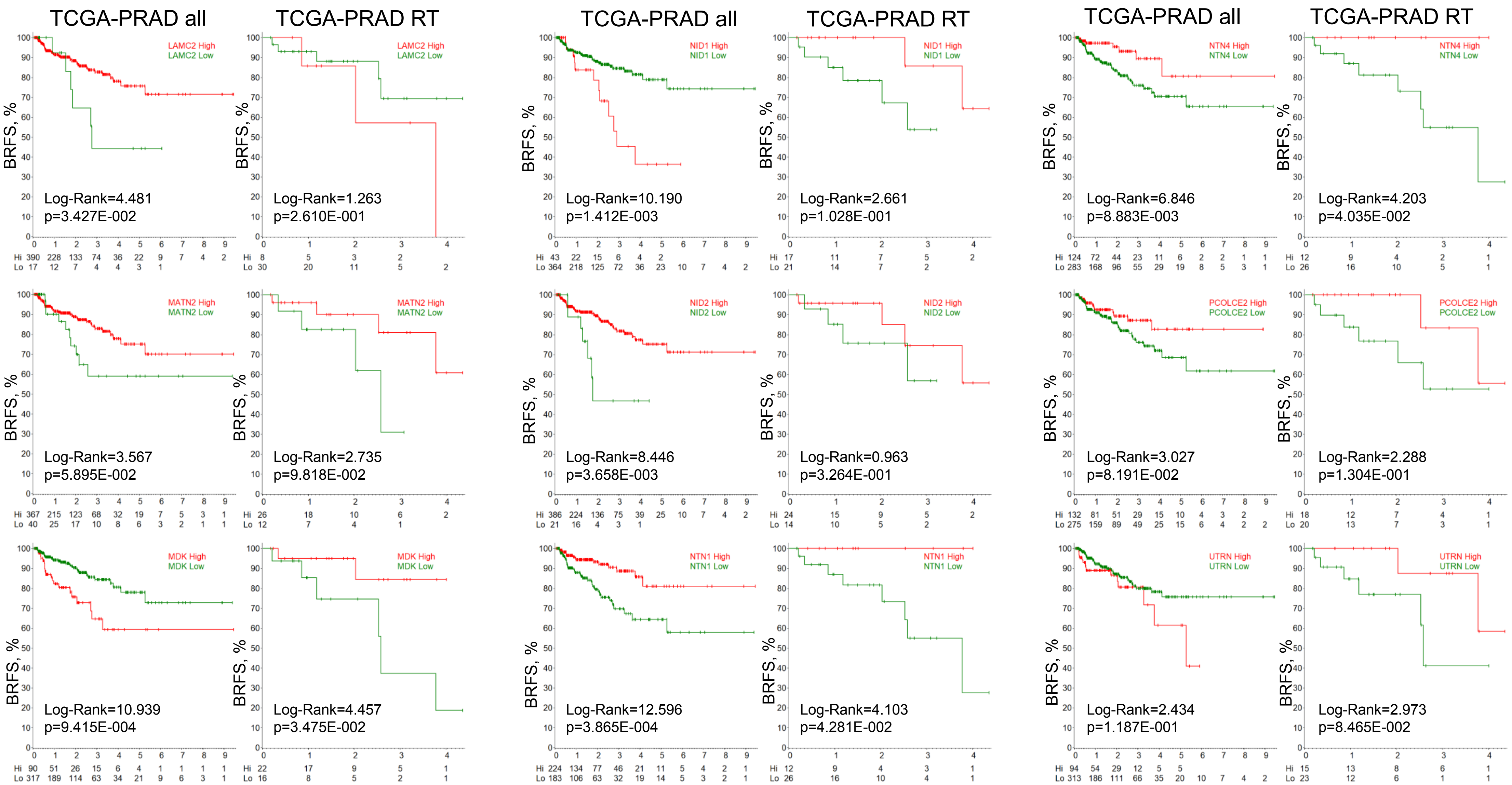

Figure S3

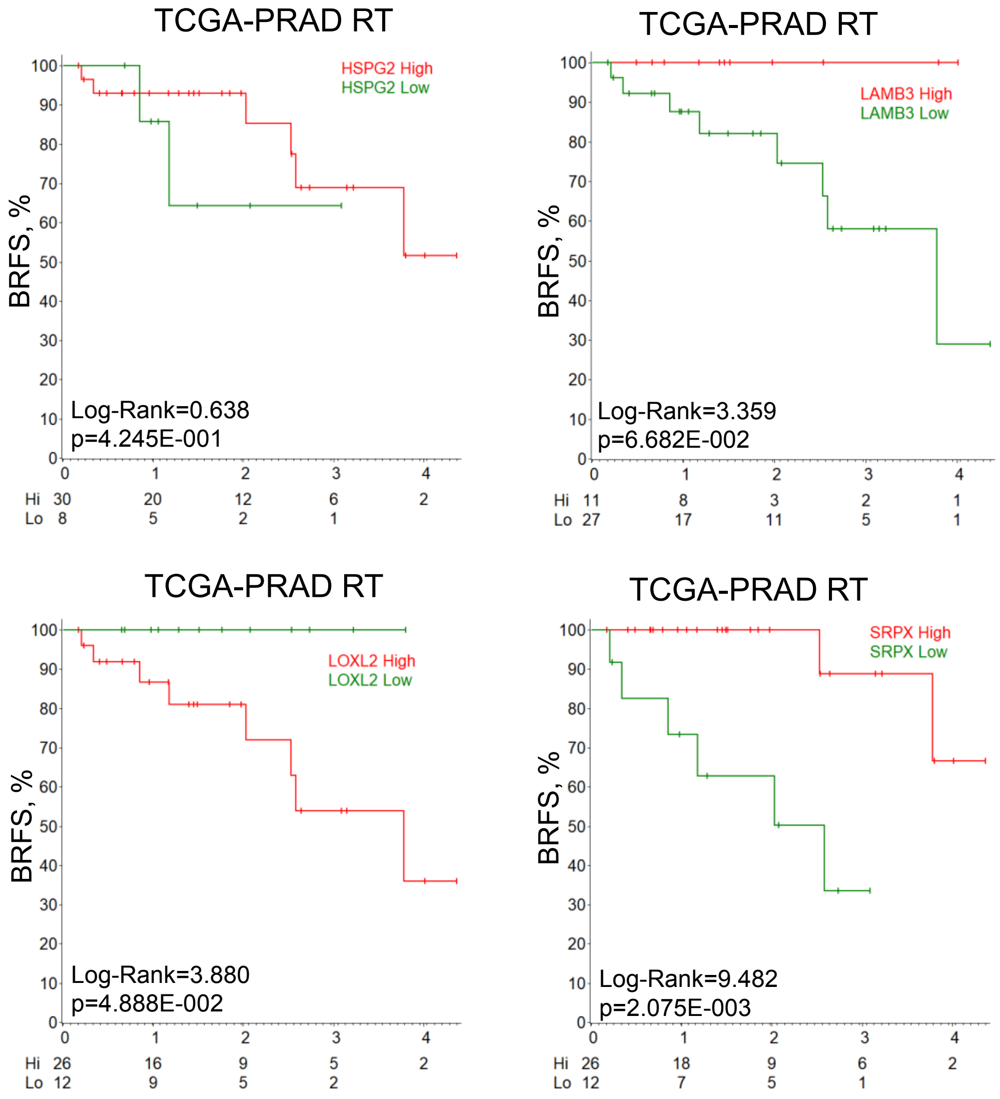

TCGA-PRAD RT
HSPG2 High
HSPG2 Low
BRFS, %
Log-Rank=0.638
p=4.245E-001
Hi 30 20 12 6 2
Lo 8 5 2 1
TCGA-PRAD RT
LAMB3 High
LAMB3 Low
BRFS, %
Log-Rank=3.359
p=6.682E-002
Hi 11 8 3 2 1
Lo 27 17 11 5 1
TCGA-PRAD RT
LOXL2 High
LOXL2 Low
BRFS, %
Log-Rank=3.880
p=4.888E-002
Hi 26 16 9 5 2
Lo 12 9 5 2
TCGA-PRAD RT
SRPX High
SRPX Low
BRFS, %
Log-Rank=9.482
p=2.075E-003
Hi 26 18 9 6 2
Lo 12 7 5 1

Figure S4

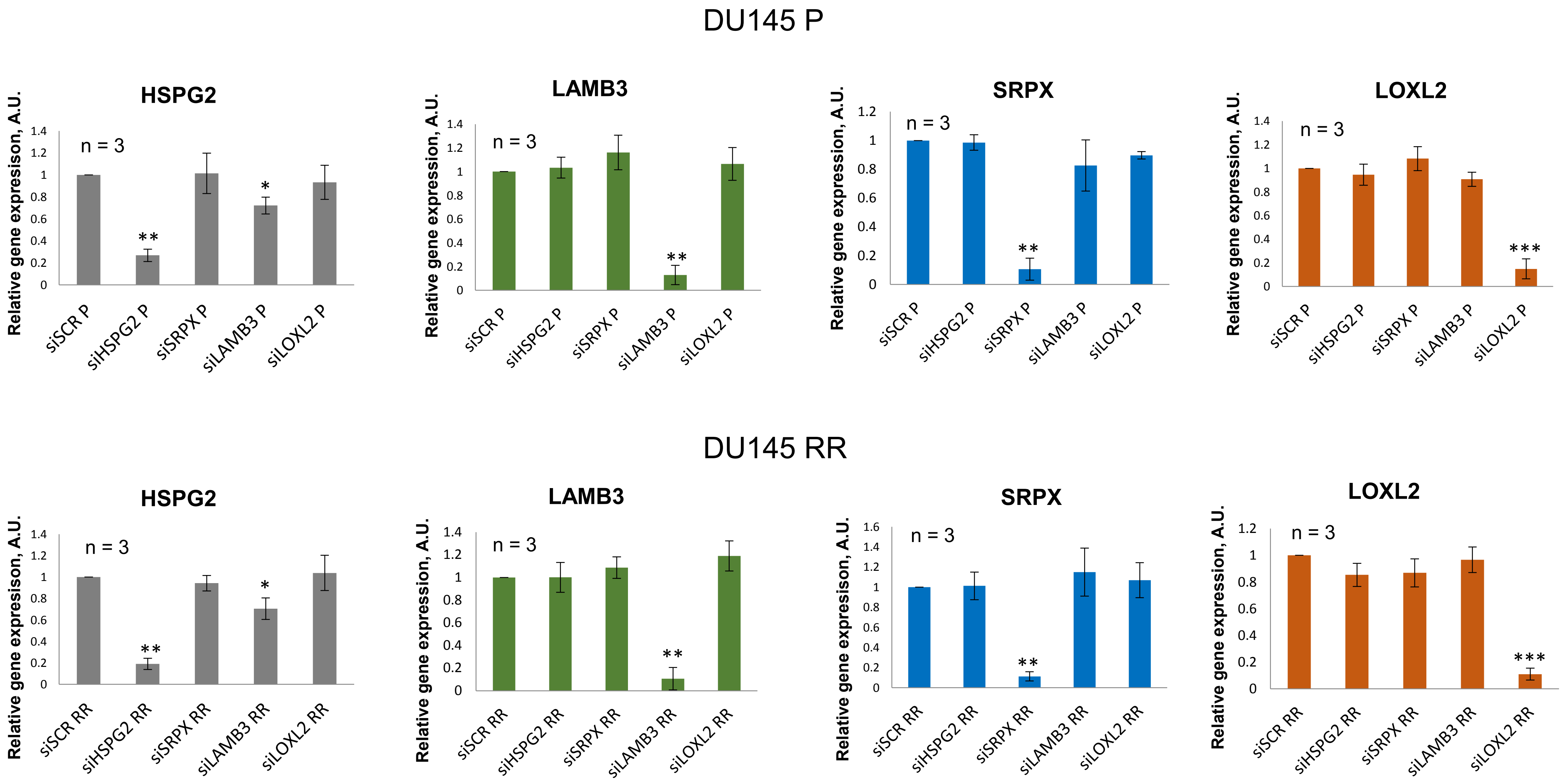
DU145 P
HSPG2
LAMB3
SRPX
LOXL2
n = 3
Relative gene expression, A.U.
siSCR P
siHSPG2 P
siSRPX P
siLAMB3 P
siLOXL2 P
DU145 RR
siSCR RR
siHSPG2 RR
siSRPX RR
siLAMB3 RR
siLOXL2 RR

Figure S5

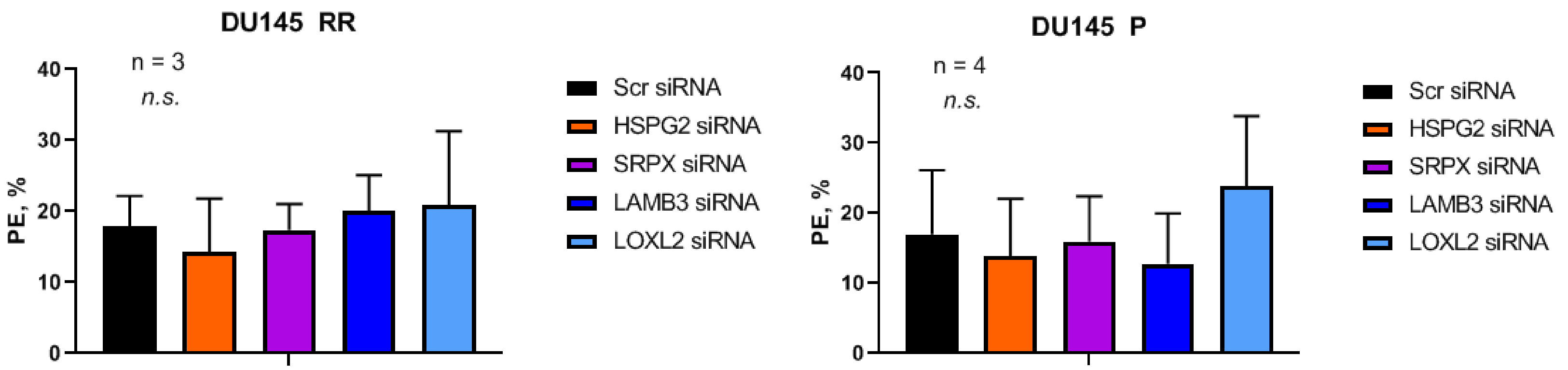
DU145 RR
n = 3
n.s.
PE, %
40
30
20
10
0
Scr siRNA
HSPG2 siRNA
SRPX siRNA
LAMB3 siRNA
LOXL2 siRNA
DU145 P
n = 4
n.s.
PE, %
40
30
20
10
0
Scr siRNA
HSPG2 siRNA
SRPX siRNA
LAMB3 siRNA
LOXL2 siRNA

Figure S6

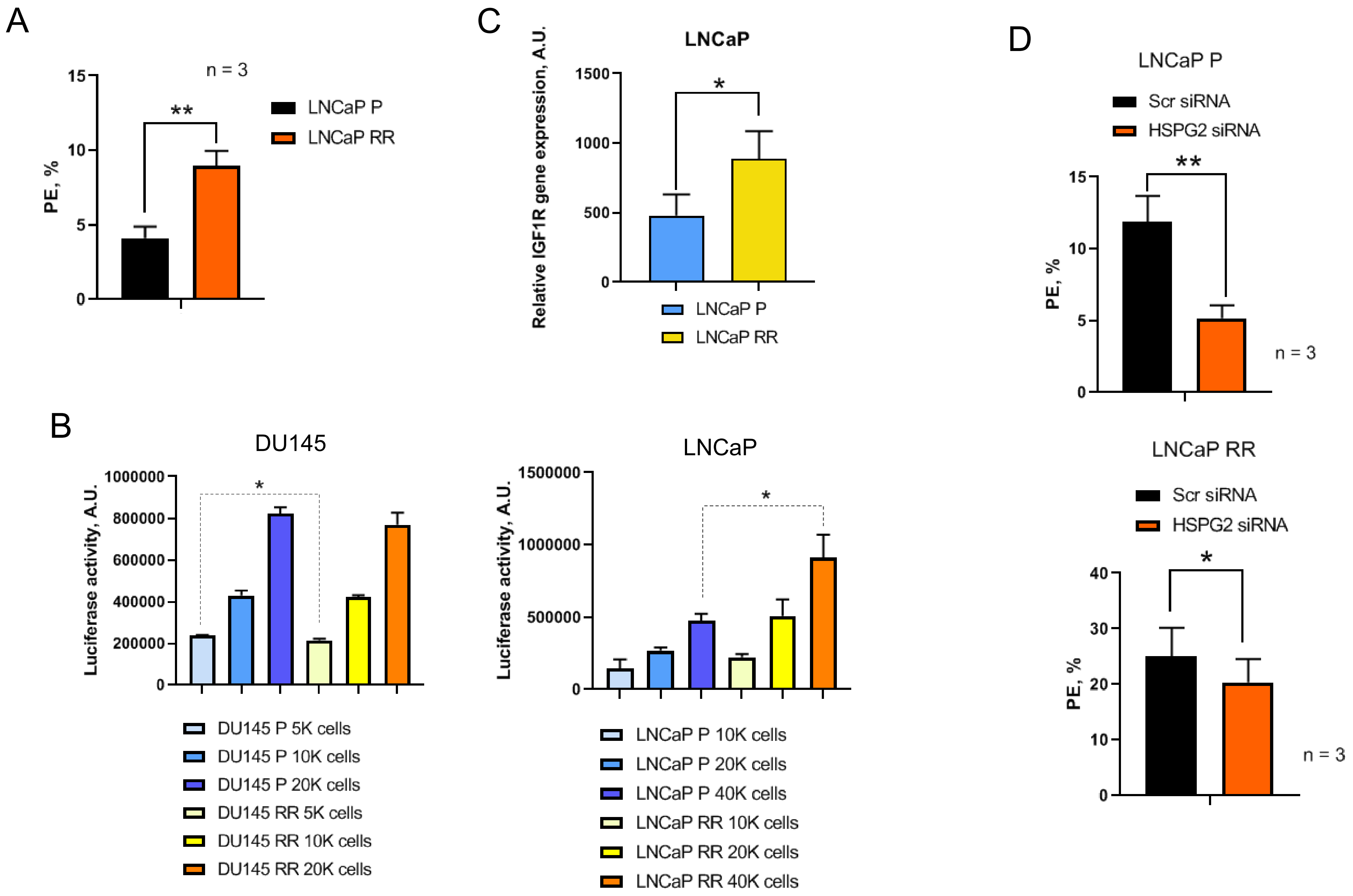

A
n = 3
**
LNCaP P
LNCaP RR
PE, %
15
10
5
0
B
DU145
*
Luciferase activity, A.U.
1000000
800000
600000
400000
200000
0
DU145 P 5K cells
DU145 P 10K cells
DU145 P 20K cells
DU145 RR 5K cells
DU145 RR 10K cells
DU145 RR 20K cells
LNCaP
1500000
1000000
500000
0
LNCaP P 10K cells
LNCaP P 20K cells
LNCaP P 40K cells
LNCaP RR 10K cells
LNCaP RR 20K cells
LNCaP RR 40K cells
C
LNCaP
Relative IGF1R gene expression, A.U.
1500
1000
500
0
LNCaP P
LNCaP RR
D
LNCaP P
Scr siRNA
HSPG2 siRNA
**
n = 3
LNCaP RR
Scr siRNA
HSPG2 siRNA
*
40
30
20
10
0
n = 3

Supplementary Discussion

Besides perlecan/HSPG2, another upregulated protein in DU145 RR cells is LOXL2, the knockdown of which did not affect the sensitivity of radioresistant DU145 RR cells. However, in DU145 P cells, LOXL2 knockdown made them resistant to irradiation. This finding is not in line with literature data that showed that LOXL2 knockdown in DU145 cells increased the sensitivity to irradiation (Xie et al., 2019). Even though LOXL2 was not identified as a target molecule in our radioresistant DU145 RR cells, there is a very extensive literature showing that the development of selective LOX inhibitors may lead to novel and effective therapies in cancer treatment because ECM stiffness regulates the expression of LOXL2 enzymes which ultimately leads to increased cancer cell proliferation and invasion (Amendola et al., 2019).

Two other proteins, SRPX and LAMB3, have a potential role in radioresistance and reduced expression, observed in DU145 RR cells. SRPX knockdown made DU145 P cells resistant to irradiation, which is in line with the decreased expression of SRPX in DU145 RR cells. However, its knockdown in DU145 RR cells unexpectedly had the opposite effect. The distinct function of SRPX in P and RR PC models can be partially explained by its role in the activation of the Ras homology family member A (RhoA) signaling pathway (Liu et al., 2019). Our previous study suggests a high activation of the RhoA pathway in DU145 RR cells, indicating its possible significance for maintaining a radioresistant phenotype in this model (Cojoc et al., 2015). However, the heterogeneous effect of SRPX knockdown on the sensitivity of PC cells to irradiation limits its potential to serve as a target in radioresistant PC. The last potential target of radioresistant PC is LAMB3, whose depletion made both cell lines, DU145 P and DU145 RR, more sensitive to irradiation than the control siRNA-transfected counterparts. However, the expression of laminin 332, a large multidomain protein encoded collectively by LAMB3, LAMA3, and LAMC2, is decreased in advanced PC (Hao et al., 2001). In PC, a protease-mediated cleavage of the β3 chain of laminin 322 encoded by LAMB3 is essential in enhancing cell motility (Tripathi et al., 2008). Therefore, LAMB3 would not be a potential target for increasing the effectiveness of anti-PC therapy.

Supplementary References:

Amendola, P. G., Reuten, R., and Erler, J. T. (2019). Interplay between LOX enzymes and integrins in the tumor microenvironment. Cancers (Basel) 11. doi: 10.3390/cancers11050729

Cojoc, M., Peitzsch, C., Kurth, I., Trautmann, F., Kunz-Schughart, L. A., Telegeev, G. D., et al. (2015). Aldehyde dehydrogenase is regulated by β-Catenin/TCF and promotes radioresistance in prostate cancer progenitor cells. Cancer Res. doi: 10.1158/0008-5472.CAN-14-1924

Hao, J., Jackson, L., Calaluce, R., McDaniel, K., Dalkin, B. L., and Nagle, R. B. (2001). Investigation into the mechanism of the loss of laminin 5 (α3β3γ2) expression in prostate cancer. American Journal of Pathology 158. doi: 10.1016/S0002-9440(10)64060-6

Liu, C. L., Pan, H. W., Torng, P. L., Fan, M. H., and Mao, T. L. (2019). SRPX and HMCN1 regulate cancer associated fibroblasts to promote the invasiveness of ovarian carcinoma. Oncol Rep 42. doi: 10.3892/or.2019.7379

Seifert M, Peitzsch C, Gorodetska I, Börner C, Klink B, Dubrovska A. (2019). Network-based analysis of prostate cancer cell lines reveals novel marker gene candidates associated with radioresistance and patient relapse. PLoS Comput Biol. 15(11):e1007460.

Tripathi, M., Nandana, S., Yamashita, H., Ganesan, R., Kirchhofer, D., and Quaranta, V. (2008). Laminin-332 is a substrate for hepsin, a protease associated with prostate cancer progression. Journal of Biological Chemistry 283. doi: 10.1074/jbc.M802312200

Xie, P., Yu, H., Wang, F., Yan, F., and He, X. (2019). Inhibition of LOXL2 Enhances the Radiosensitivity of Castration-Resistant Prostate Cancer Cells Associated with the Reversal of the EMT Process. Biomed Res Int 2019. doi: 10.1155/2019/4012590
